# Minipig costal and knee cartilage structure-function relationships and their use as cell sources for tissue-engineered analogous cellular products for cartilage repair

**DOI:** 10.1016/j.actbio.2025.12.023

**Published:** 2025-12-11

**Authors:** Gaston A. Otarola, Rachel C. Nordberg, Jerry C. Hu, Kyriacos A. Athanasiou

**Affiliations:** Department of Biomedical Engineering, 3131 Engineering Hall, University of California, Irvine, CA 92617, USA

**Keywords:** Cartilage, Tissue engineering, Orthopedic biomaterials, Regenerative medicine, Clinical translation, Structure-function relationships

## Abstract

Toward the development of biologic products intended to repair articular cartilage, this study evaluated native structure-function relationships of rib and knee cartilage and compared the capacity of articular chondrocytes (ACs) and costochondral cells (CCs) to generate tissue-engineered analogous cellular products (ACPs) for preclinical cartilage repair studies. Utilizing the relevant preclinical model of the Yucatan minipig, both knee and rib cartilages were characterized by topography and age. Key findings include that: 1) knee cartilage had higher tensile properties than rib cartilage (e.g., ultimate tensile strength of adult knee cartilage being 218 % of adult rib cartilage), 2) rib cartilage had higher compressive properties than knee cartilage (e.g., aggregate modulus of adult rib cartilage being 177 % of adult knee cartilage), 3) functional (i.e., biomechanical and biochemical) properties were dependent on age (e.g., adult coefficient of friction being 186 % of that of the juvenile in the medial-lateral axis of the knee), 4) functional properties were dependent on topography, 5) compressive properties were significantly correlated to glycosaminoglycan content and hydration, and 6) tensile properties were significantly correlated to collagen content. Additionally, juvenile ACs and CCs were compared for their capacity to generate self-assembled neocartilage with constructs achieving functionality index values (i.e., a weighted average of neocartilage functional properties compared to native tissue) of 0.54 and 0.44, respectively, when compared to native adult minipig knee cartilage. Overall, this study provides gold-standard characterization values for minipigs of different ages for preclinical cartilage repair studies and demonstrates that both ACs and CCs can be used to generate functional self-assembled neocartilage ACPs.

**Statement of significance:**

Toward the clinical translation of tissue-engineered cartilage implants, this study characterizes the functional properties of knee and rib cartilage in the clinically relevant Yucatan minipig model. Additionally, it evaluates the ability of knee- and rib-derived chondrocytes to generate neocartilage that recapitulates the functional properties of native cartilage tissues. Age-related changes in rib and knee cartilage are also described, along with structure-function relationships involving correlating biochemical composition and mechanical properties of cartilage tissues. Overall, this study provides data essential to the translation of cartilage implants for the knee and describes how these data are relevant to the FDA regulatory process.

## 1. Introduction

Allogeneic costal cartilage is a valuable source of chondrocytes for developing new therapies to treat chondral injuries in the form of tissue-engineered, off-the-shelf implants [1,2]. Current cartilage repair strategies, such as osteochondral autograft transplantation (OATS), minimally manipulated allografts (e.g., Cartiform, ProChodnrix, DeNovo NT), and autologous cellularized products (e.g., MACI), fail to restore the native tissue or provide long-term restoration [3–6]. Self-assembled neocartilage is under evaluation as a promising strategy for cartilage repair, which is a tissue-engineered approach that does not employ an exogenous scaffold and is capable of recapitulating native cartilage structure and functionality (e.g., biomechanical properties), biochemistry, and mechanics using expanded chondrocytes [7,8]. Self-assembled neocartilage has been generated using techniques that preserve the chondrogenic phenotype during expansion, [9] which enables the use of cell bank systems for the consistent production of allogeneic cartilage implants from a single validated cell source [10]. Additionally, self-assembled neocartilage implants made with costochondral cells (CCs, including costal chondrocytes and chondroprogenitor cells) [11] obtained from ribs have been used to successfully regenerate the temporomandibular joint disc; [12] these *in vivo* results hold promise for eventual clinical translation given that the U.S. Food and Drug Administration (FDA) requires preclinical studies in large animal models (e.g., the minipig) in their guidance documents [13]. Self-assembled minipig CC neocartilage has been generated that recapitulates the functional properties of human knee cartilage (i.e., 600–880 kPa aggregate modulus and 3–12 MPa Young’s modulus in minipig CC neocartilage [14] vs. 380–780 kPa aggregate modulus and 7–33 MPa Young’s modulus in the human knee [15]). Despite these advances, the functional properties of costal cartilage compared to those of articular cartilage and the differences between AC- and CC-derived neocartilages remain unclear.

Toward translating self-assembled neocartilage to clinical use, the FDA regulatory process requires completion of preclinical animal studies that demonstrate the safety and efficacy of the product [10,16,17]. Despite the regulatory burden, a neocartilage implant derived from banked allogeneic chondrocytes carries multiple advantages; well-characterized cell banks can be used to fabricate large quantities of product on demand, in a reproducible manner, with known functional properties, free of diseases, and with little biological variability [10]. Overall, cell banks following the eliminate issues associated with autologous cell-based therapies such as an initial harvest surgery, the potential of donor site morbidity, issues related to the donor age, and quality of the autologous donor cartilage [10,16]. Toward the establishment of tissue-engineered implants for cartilage repair, one would consider the characteristics of the donor tissue and whether the cells are capable of forming implants with properties similar to the target tissue.

For the translation of cartilage and other products (e.g., cardiovascular), the Yucatan minipig is an excellent preclinical model due to its anatomy, physiology, and temperament [13,17,18]. These characteristics facilitate the establishment of surgical approaches, defect parameters, implant fixation techniques, and rehabilitation protocols to increase the overall chances of success [13]. For human cells, tissues, and cellular and tissue-based products (HCT/Ps), the FDA has described the use of analogous cellular products (ACPs,) defined as “cellular products derived from the animal species used for testing that are analogs of the ultimate clinical product in phenotype and biologic activity” [19]. Toward comparing tissue-engineered ACPs with the functional properties of native tissue, a functionality index has been developed, [20–22] which is a weighted average of salient functional properties of the implant compared against those of the intended tissue to be repaired. Thus, in addition to comparing individual properties, the functionality index allows for a comprehensive view of how analogous an ACP is to the HCT/P. An ACP needs to be sufficiently analogous to the HCT/P to be released for use in preclinical testing. Despite achieving minipig-derived self-assembled neocartilage properties that resemble those of native human cartilage, whether minipig ACPs can satisfy functionality index-based release criteria remains unclear because the field still lacks the functional values of native minipig knee and rib cartilages, necessitating the characterization of these tissues.

Characterizing the biomechanical properties of minipig cartilages, including age-dependent and topographical variations, will provide data toward the planning and interpretation of preclinical studies. For example, adult minipig cartilage has been reported to have different mechanical properties from juvenile cartilage, such as lower instantaneous modulus values in the minipig acetabulum [23]. To date, no comprehensive characterization of the knee or rib cartilages of minipig with respect to age has been performed. Moreover, existing literature strongly suggests that topographical variations of cartilage properties, especially in the knee, can impact study outcomes. For example, the mechanical properties of articular cartilage differ throughout the topography of the knee; in the sheep, tensile Young’s modulus and ultimate tensile strength (UTS) values were found to be lower in the posterior medial condyle when compared to the anterior region [1]. These differences highlight the importance of how variations due to age and topography can affect tissue engineering strategies and preclinical studies.

This study characterized costal cartilage and knee articular cartilage of the Yucatan minipig animal model, interrogated the structure-function relationships of these tissues, and evaluated the use of CCs and ACs for articular cartilage tissue engineering. These two cartilage locations were selected because 1) articular cartilage of the knee is a common source of clinical morbidity but has yet to be characterized in the Yucatan minipig animal model, and 2) costal cartilage has been used to source chondrocytes for cartilage repair, [12] but there has not been a comprehensive characterization of the tissues in the minipig. Evaluating multiple cartilage types within a single study with consistent testing and reporting conditions minimizes interstudy variability, thereby aiding the elucidation of structure-function relationships not apparent in single-tissue analyses. The overall hypotheses of this study were that 1) tissue properties would differ based on anatomical structure (i.e., rib vs. knee), topographical location, and age and 2) both AC- and CC-derived neocartilages would achieve appropriate release criteria as ACPs.

## 2. Methods

### 2.1. Study design

Two age groups (juvenile and adult) were selected because juvenile cartilage tissue has been identified as a preferred cell source for cartilage tissue engineering, [24] but patients who undergo cartilage repair procedures are typically adults whose tissues have different properties [25]. As part of the experimental design, these tissues were characterized by topographical location; i.e., the medial condyle, lateral condyle, and trochlear groove of knee articular cartilage, and the medial and lateral aspects of costal cartilage. In addition to tissue-level characterizations, juvenile AC- and CC-derived neocartilages were tissue-engineered and compared for their capacity to generate self-assembled neocartilage. To elucidate structure-function relationships of the native tissues, Pearson correlations were used to relate biochemical compositions to mechanical properties. Then, using data derived from the characterization of the native tissues, FIs were calculated to determine how well AC- and CC-derived neocartilages recapitulate the function of young and adult native tissues.

#### 2.1.1. Sample collection

**Fig. 1.**
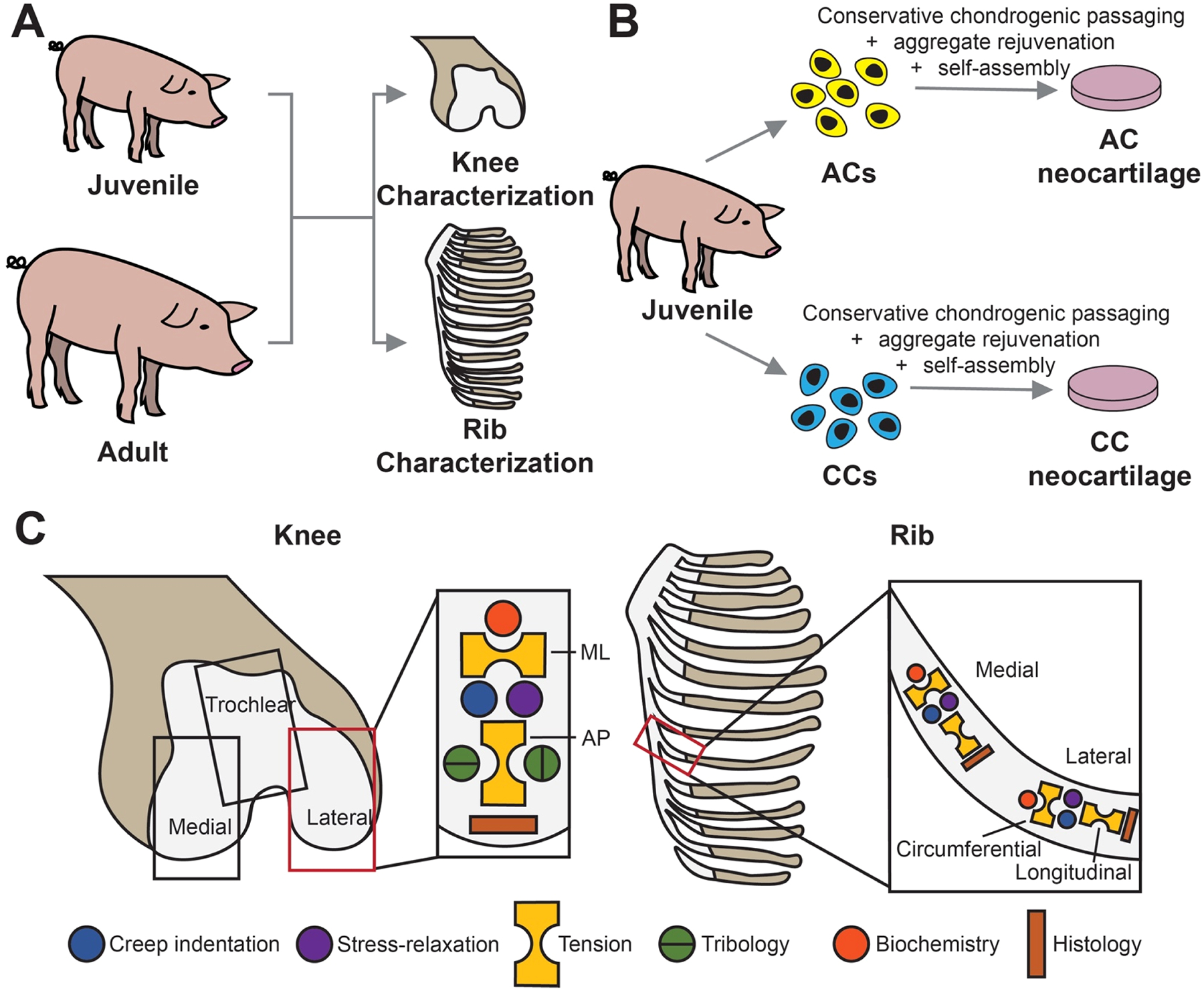
Experimental overview. A. Tissue was isolated from the rib and knee of juvenile and adult minipigs. B. In addition, cells were isolated from the juvenile tissue to generate self-assembled neocartilage. C. For characterization studies, samples were collected from the medial condyle, lateral condyle, and trochlear groove in the knee and from the medial and lateral aspects of the rib. Creep indentation samples, stress-relaxation samples, tensile samples (two orientations), tribological samples (two orientations), biochemical samples, and histological samples were isolated as depicted in the diagram. For the testing that was conducted in two orientations, orientations were defined as knee = anterior-posterior (AP) axis and medial-lateral (ML) axis, rib = longitudinal and circumferential.

As depicted in Fig. 1A, porcine articular and rib cartilage samples were collected from a total of seven juvenile minipigs (5–7 months; four males, three females) and eight skeletally mature minipigs (>18 months; four males, four females) from Premier BioSource (California, USA). This work was exempt because no live animals were used, and the tissues were obtained from animals culled for reasons unrelated to this study. Rib costochondral tissue was obtained from the lateral and medial aspects of rib number 11. Knee articular cartilage was obtained from the medial condyle, the lateral condyle, and the trochlear groove. To ensure balanced sampling, tissue was harvested alternately from the right and left sides (i.e., 3–4 per side for each anatomical structure and age group), with each animal serving as an independent biological replicate (*n* = 7–8 animals per group). Samples collected from each region were cut, and labeled as follows: (a) 5 mm diameter samples for histology, (b) 2 mm by 2 mm samples for biochemical analyses, (c) dog bone-shaped pieces of tissue were taken from two orientations for tensile testing (orientations were defined as knee = anterior-posterior (AP) and medial-lateral (ML), rib = longitudinal and circumferential), (d) 3 mm diameter samples were used for compressive testing, (e) 5 mm diameter samples with orientation marked were used for tribology (Fig. 1B). Samples were fixed in formalin for histology; all other samples were frozen at −20 °C in phosphate-buffered saline, for biochemical testing, or protease inhibitor solution (NaCl 8.766 g, EDTA 0.673 g, benzamidine HCl, 0.783 g, N-ethylmaleimide 1.251 g, and phenylmethylsulfonylfluoride 0.174 g per liter of water) until testing, for mechanical testing. Samples were thawed only for testing to avoid any changes in outcome measures [26].

#### 2.1.2. Isolation and expansion of articular chondrocytes and costochondral cells

Juvenile porcine ACs and CCs were harvested from four juvenile Yucatan minipigs (2 males, 2 females) (Premier BioSource, California, USA) no later than 48 h after being culled. ACs were isolated from the medial condyle, lateral condyle, and trochlear groove. CCs were isolated from the unmineralized portion of costal cartilage from all ribs. The cartilages were minced to 1 mm^3^, washed three times with GlutaMAX Dulbecco’s Modified Eagle Medium containing 4.5 g/L glucose (DMEM; Gibco) and 1 % (v/v) penicillin/streptomycin/fungizone (PSF; Lonza, Basel, Switzerland), and digested with 0.4 % pronase (Sigma) in DMEM for 1 h at 37 °C, and then in 0.2 % collagenase type II (Worthington Biochemical, Lakewood, NJ) in DMEM supplemented with 3 % (v/v) fetal bovine serum (FBS; Atlanta Biologicals, Lawrenceville, GA) for 18 h at 37 °C. Cells were strained through a 70 μm strainer and washed with ACK buffer for 4 min, as previously described [27]. Primary (P0) CCs were seeded in T225 flasks at a density of ~2.5 million cells per flask in chondrogenic medium (CHG) consisting of DMEM, 1 % (v/v) PSF, 1 % (v/v) insulin-transferrin-selenium (BD Biosciences, San Jose, CA), 1 % (v/v) non-essential amino acids (Thermo Fisher Scientific), 100 mg/mL sodium pyruvate (Thermo Fischer Scientific), 50 mg/mL ascorbate-2-phosphate (Sigma, St. Louis, MO), 40 mg/mL L-proline (Sigma), 100 nM dexamethasone (Sigma), 2 % (v/v) FBS, 1 ng/mL TGF-β1, 5 ng/mL bFGF, and 10 ng/mL PDGF (PeproTech). After passage 1 (P1), the cells were then counted and frozen in medium containing 90 % (v/v) FBS + 10 % (v/v) DMSO (Sigma) until further expansion. To generate constructs, cells were thawed and passaged to P3 at confluence using 0.05 % trypsin-EDTA (Invitrogen) and then 0.2 % collagenase type II and 3 % FBS in DMEM. All cell culture occurred at 37 °C in 10 % CO_2_.

#### 2.1.3. Chondrogenic differentiation in aggregate rejuvenation and neocartilage construct seeding

Aggregate rejuvenation was performed to recover the chondrogenic phenotype of expanded chondrocytes as previously described [9]. Briefly, cells were seeded on 1 % (w/v) agarose-coated plates at a density of 750,000 cells/mL (30 mL total) per plate with CHG and 10 ng/ml TGF-β1, 100 ng/ml GDF-5, and 100 ng/ml BMP-2 (PeproTech) [9]. Plates were placed on an orbital shaker at 50 rpm for 24 h and further cultured statically for 14 days with medium changes every 4 days. Aggregates were digested using 0.05 % trypsin-EDTA and then 0.2 % collagenase type II and 3 % FBS in DMEM. After passing through a 70 μm cell strainer, cells were washed twice, resuspended in CHG, and self-assembled into neocartilage by seeding 2 million cells in 5 mm diameter 2 % (w/v) agarose wells at a density of 20 million cells/ml. At 4 h after seeding, 400 μL of CHG supplemented with 10 ng/ml TGF-β1 was added. Medium was replaced daily until unconfinement (at day 3 post self-assembly), after which, constructs were kept in 2 mL of medium, replaced every 2 days. TGF-β1 was applied at 10 ng/ml after seeding and until day 28, c-ABC (Sigma) was applied at 2 Units per mL for 4 h on day 7, and LOXL2 (Signal Chem) was applied at 0.15 μg/mL between days 7 and 21 after seeding, together with 0.146 μg/mL hydroxylysine (Sigma) and 1.6 μg/mL copper sulfate (Sigma). Constructs were tested after 28 days of culture.

#### 2.1.4. Histology

Native cartilage and neocartilage samples were fixed in 10 % neutral-buffered formalin. Prior to embedding, rib samples were decalcified in 20 % EDTA for 2 weeks. Paraffin-embedded samples were sectioned to a thickness of 6 μm, cleared, and stained with hematoxylin and eosin (H&E), safranin-O and fast green, and picrosirius red using standard protocols.

#### 2.1.5. Biochemical properties

Samples were weighed for wet weight (WW), lyophilized for 3 days, and weighed for dry weight (DW). Then, samples were digested in 125 μg/mL papain (Sigma), 5 mM N-acetyl-l-cysteine, and 5 mM EDTA in phosphate buffer pH 6.5 for 18 h at 60 °C. Total collagen was quantified using a modified chloramine-T hydroxyproline assay using Sircol 0.5 mg/ml acid-soluble bovine collagen as a standard (Biocolor, Newtownabbey, Northern Ireland) [20]. Sulfated GAG content was quantified using a Blyscan Glycosaminoglycan Assay kit (Biocolor), and DNA content was quantified using Picogreen (ThermoFisher Scientific) following manufacturers’ protocols.

#### 2.1.6. Tribological properties

A custom pin-on-disk tribometer was used to assess the tribology of minipig cartilage under boundary lubrication conditions, as described previously [28]. For native tissue, measurements parallel and perpendicular to the direction of articulation were taken using 3 mm punches, trimmed to a ~1 mm height. Unlike native tissue with alignment, neocartilage constructs were measured in only one direction. Samples were immersed in phosphate-buffered saline (PBS) for 2 min to reach equilibrium and then sheared against the test surface for 5 min with velocity set to 0.5 mm/s and a compressive normal force applied by a 200 g mass (native tissue) or a 50 g mass (neocartilage). The resultant applied stress corresponds to 0.28 MPa for native tissues and 0.07 MPa for neocartilage tissues, as described previously [15,28]. The measured coefficient of friction describes the interaction between the articular cartilage sample and the underlying glass plate immersed in PBS. Data were collected at a rate of 50 Hz.

#### 2.1.7. Tensile properties

Uniaxial tensile testing was conducted using an Instron model 5565 (Instron, Canton, MA). Dog bone-shaped samples were collected from all native tissue and neocartilage samples with gauge lengths ranging from 0.8 to 1.55 mm. For knee articular cartilage, samples were collected both parallel and perpendicular to the direction of articulation (i.e., the anterior-posterior axis and medial-lateral axis, respectively). For rib cartilage, samples were tested along the length and width of the rib (i.e., longitudinally and circumferentially, respectively). All samples were prepared and photographed to measure thickness and width using ImageJ, with an average cross-sectional area of 0.248 mm^2^ ± 0.023mm^2^ and pulled at a strain rate of 1 % of the gauge length/second until failure. Samples that failed to break in the midportion were excluded from the data set. The Young’s modulus was defined as the slope of the linear region of the stress-strain curve, ultimate tensile strength (UTS) was defined as the maximum stress obtained, strain-at-failure was defined as the strain when the ultimate tensile stress was measured, resilience was defined as the area under the curve (energy) until permanent deformation, and toughness was defined as the area under the curve (energy) until fracture.

#### 2.1.8. Compressive properties

Both creep indentation and stress-relaxation methodologies were employed to determine compressive properties. Briefly, 3 mm punches were obtained from all native tissue and neocartilage samples and trimmed to a height of 1 mm. For creep indentation, samples were tested on a custom-made creep indentation apparatus (CIA) [29]. Each sample was photographed, then submerged in PBS until equilibrium, and indented with a flat porous 1 mm diameter tip perpendicular to the surface of the sample. A tare mass of 0.2 g was applied until equilibrium was achieved, and a test mass of 2.2–12.5 g was applied during testing, which corresponded to ~10 % strain, depending on the sample. A biphasic model and finite-element optimization were then used to fit the raw data for determining the aggregate modulus, shear modulus, and permeability values [30]. For stress-relaxation, samples were tested using an Instron model 5565 (Instron, Canton, MA). First, the sample height was determined by compressing the sample at a rate of 0.025 mm/s until reaching a 0.2 N force. For testing, samples were preconditioned with 10 cycles at 5 % strain and a 10 % strain rate of the sample height/second, and incremental stress-relaxation was performed at 10 % and 20 % strain. That is, samples were compressed to 10 % strain, allowed to relax to equilibrium, compressed to 20 % strain, and allowed to once more relax to equilibrium. The 10 % relaxation modulus, 10 % instantaneous modulus, 20 % relaxation modulus, and 20 % instantaneous modulus were then determined using a standard linear solid viscoelasticity model [31].

#### 2.1.9. Functionality index

The degree to which neocartilage recapitulated functional properties of native adult knee cartilage (i.e., the intended tissue to be repaired clinically) was quantified using a functionality index [20–22]. The functionality index equation is a weighted average of salient functional properties of engineered tissue compared to native tissue (Eq. (1)). The more similar the properties of neocartilage are to those of native tissue, the closer the functionality index value is to 1. In this equation, E^C^ is the aggregate modulus, E^T^ is the tensile Young’s modulus, CoF is the coefficient of friction, Col is collagen/WW, and GAG is glycosaminoglycan/WW for native (Nat) and engineered (Eng) tissue. To apply Eq. (1), the corresponding functional values (e.g., tensile Young’s modulus for E^T^) from the native tissue characterization and tissue-engineered constructs are substituted into the equation. In this study, the functionality index was calculated both for the knee as a whole (i.e., averaging values from the native medial condyle, lateral condyle, and trochlear groove) and specifically for the medial condyle, which is the site most commonly reconstructed in clinical practice [32].


(1) $$FI=\frac{1}{5}\left[\left(1-|\frac{E_{Nat}^{C}-E_{Eng}^{C}}{E_{Nat}^{C}}|\right)+\left(1-|\frac{E_{Nat}^{T}-E_{Eng}^{T}}{E_{Nat}^{T}}|\right)\;+\left(1-|\frac{{\text{CoF}}_{\text{Nat}}{-\text{CoF}}_{\text{Eng}}}{{\text{CoF}}_{\text{Nat}}}|\right)+\left(1-|\frac{{\text{Col}}_{\text{Nat}}{-\text{Col}}_{\text{Eng}}}{{\text{Col}}_{\text{Nat}}}|\right)\;+\left(1-|\frac{{\text{GAG}}_{\text{Nat}}{-\text{GAG}}_{\text{Eng}}}{{\text{GAG}}_{\text{Nat}}}|\right)\right]$$

#### 2.1.10. Statistical analysis

**Fig. 2.**
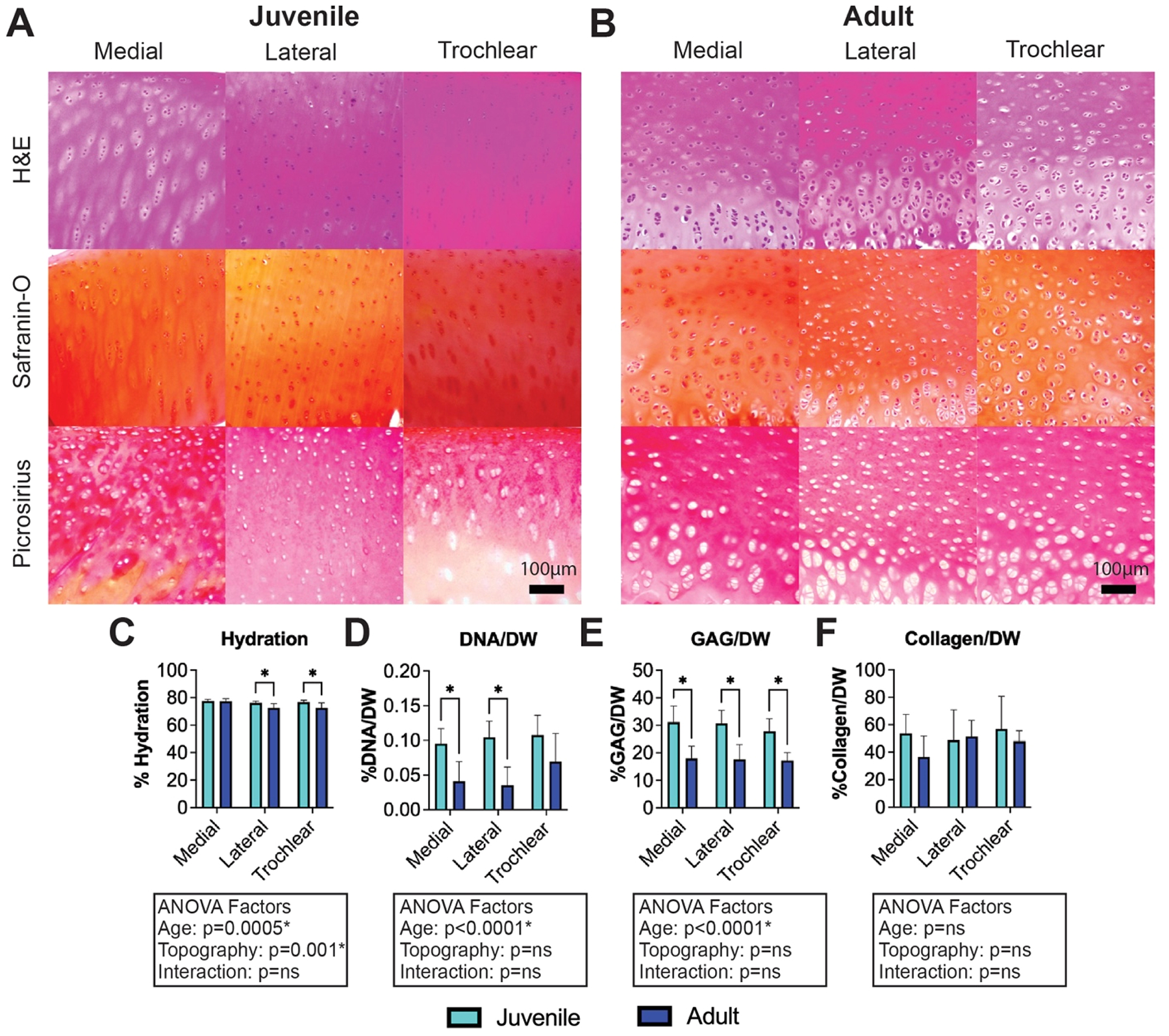
Knee histology and biochemistry. Histological sections were taken from the medial condyle, lateral condyle, and trochlear groove of juvenile and adult knee tissue (A-B). Tissue was stained using Hematoxylin and Eosin, Safranin-O, Picrosirius Red (scale bar = 100 μm). Biochemical contents of each tissue group were quantified by hydration, DNA/DW, GAG/DW, Collagen/DW (C-F). Values presented are mean+standard deviation for *n* = 7–8. Statistical analyses were conducted through a two-way ANOVA followed by juvenile and adult comparisons using *t*-tests with a Holm-Sidak correction for multiple comparisons (*p < 0.05).

**Fig. 3.**
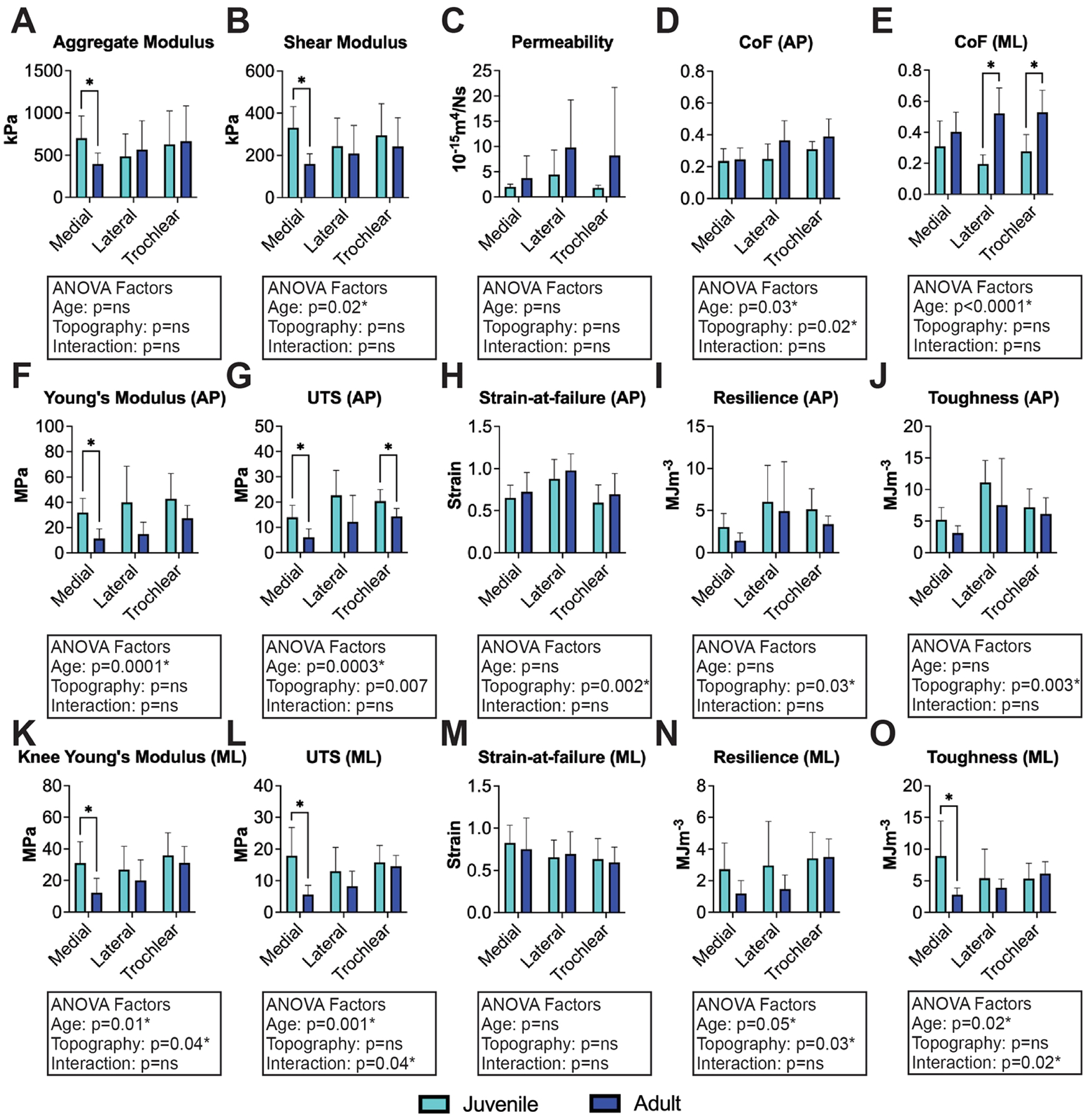
Knee mechanical properties. Mechanical samples were taken from the medial condyle, lateral condyle, and trochlear groove of juvenile and adult knee tissue, and compressive (A-C), tribological (D-E), and tensile (F-O) properties were assessed. Values presented are mean+standard deviation for *n* = 6–8. Statistical analyses were conducted through a two-way ANOVA followed by juvenile and adult comparisons using *t*-tests with a Holm-Sidak correction for multiple comparisons (*p < 0.05). AP = anterior-posterior axis, ML = medial-lateral axis.

**Fig. 4.**
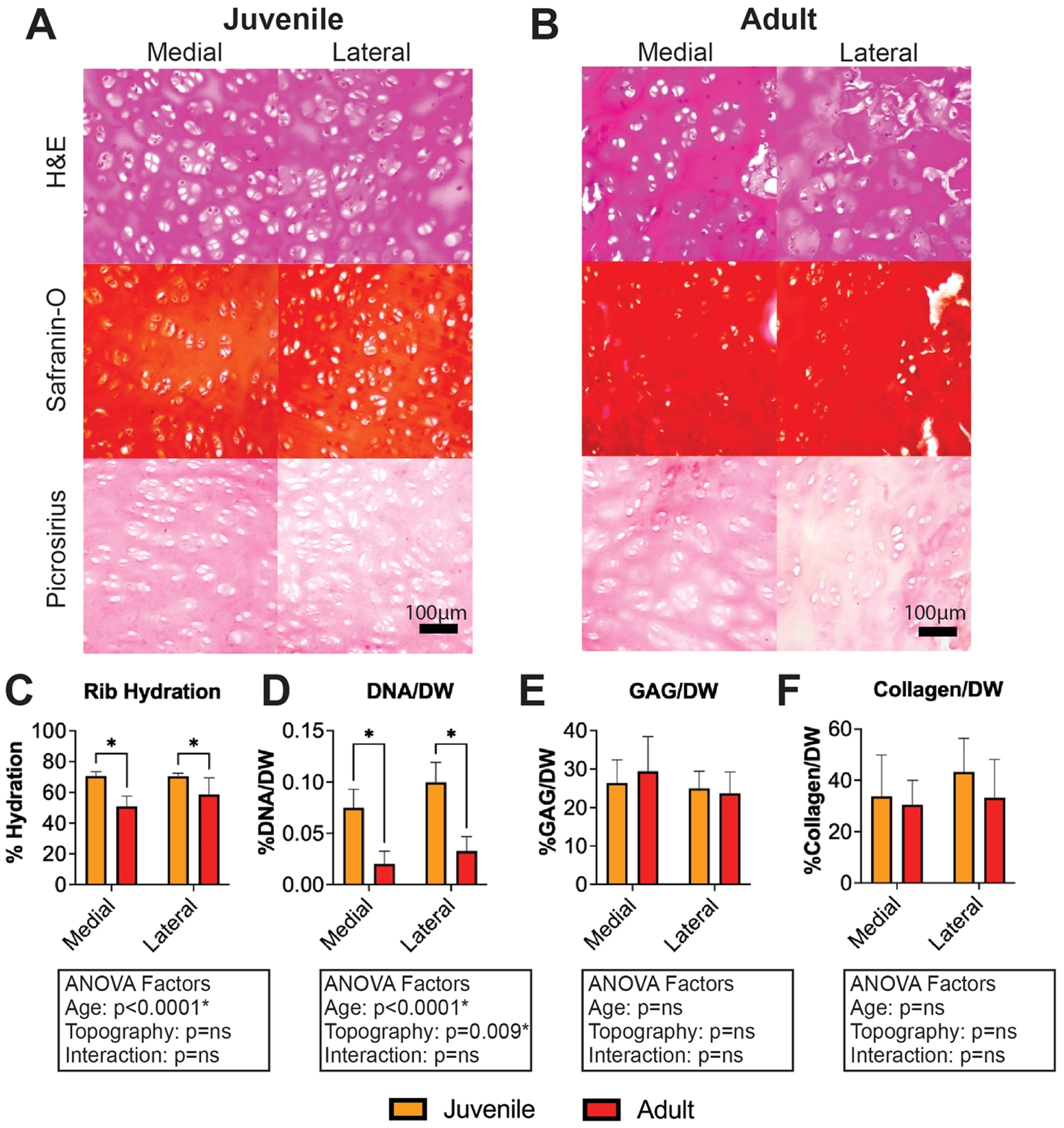
Rib histology and biochemistry. Histological sections were taken from the medial and lateral aspects of juvenile and adult rib tissue (A-B). Tissue was stained using Hematoxylin and Eosin, Safranin-O, Picrosirius Red (scale bar = 100 μm). Biochemical contents of each tissue group were quantified by hydration, DNA/DW, GAG/DW, Collagen/DW (C-F). Values presented are mean+standard deviation for *n* = 6–8. Statistical analyses were conducted through a two-way ANOVA followed by juvenile and adult comparisons using *t*-tests with a Holm-Sidak correction for multiple comparisons (*p < 0.05).

**Fig. 5.**
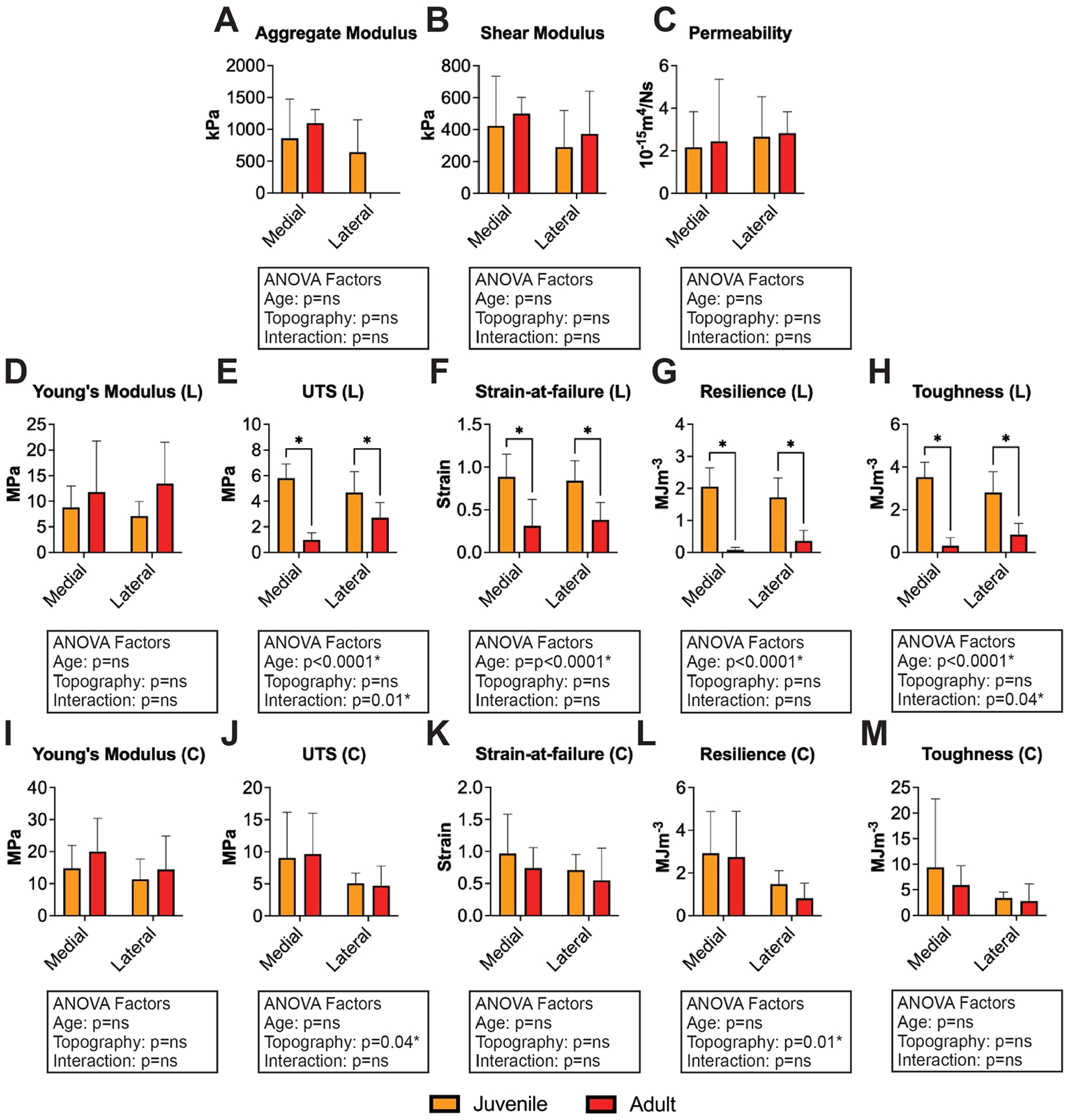
Rib mechanics. Mechanical samples were taken from the medial and lateral aspects of juvenile and adult rib tissue, and compressive (A-C) and tensile (D-M) properties were assessed. Values presented are mean+standard deviation for *n* = 6–8. Statistical analyses were conducted through a two-way ANOVA followed by juvenile and adult comparisons using *t*-tests with a Holm-Sidak correction for multiple comparisons (*p < 0.05). *L* = longitudinal axis, *C*= circumferential axis.

**Fig. 6.**
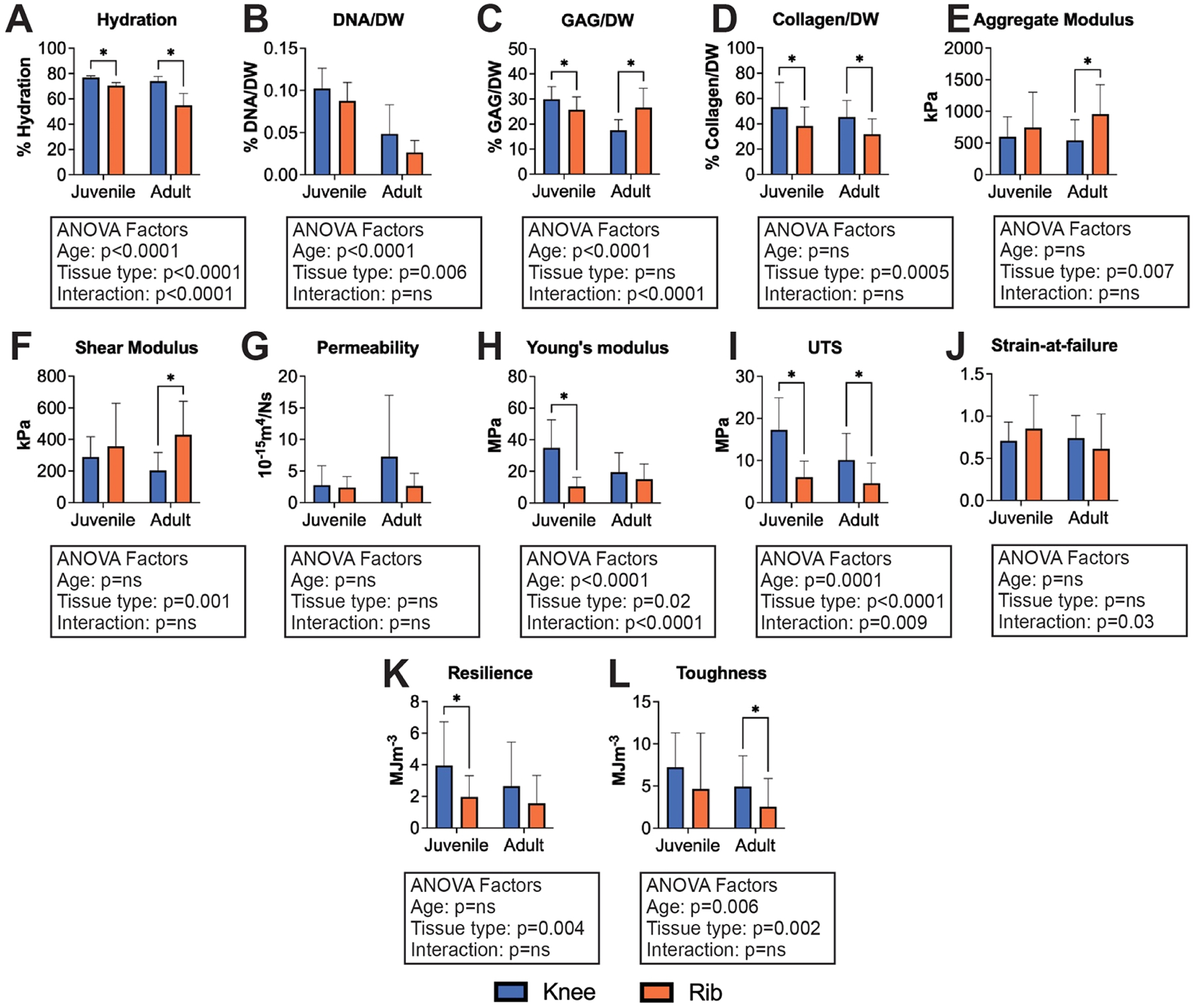
Summary of tissue-specific (i.e., knee vs. rib) and age-specific (i.e., juvenile vs. adult) trends. Biochemical (A-D), compressive (E-G), and tensile (H-L) data are presented. All topographical data points were pooled to obtain an overall representation for each tissue type (i.e. juvenile rib, adult rib, juvenile knee, adult knee). Values presented are mean+standard deviation. Statistical analyses were conducted through a two-way ANOVA followed by *t*-tests with a Holm-Sidak correction for multiple comparisons to assess differences between anatomical structure (i.e., knee vs. rib) (*p < 0.05).

**Fig. 7.**
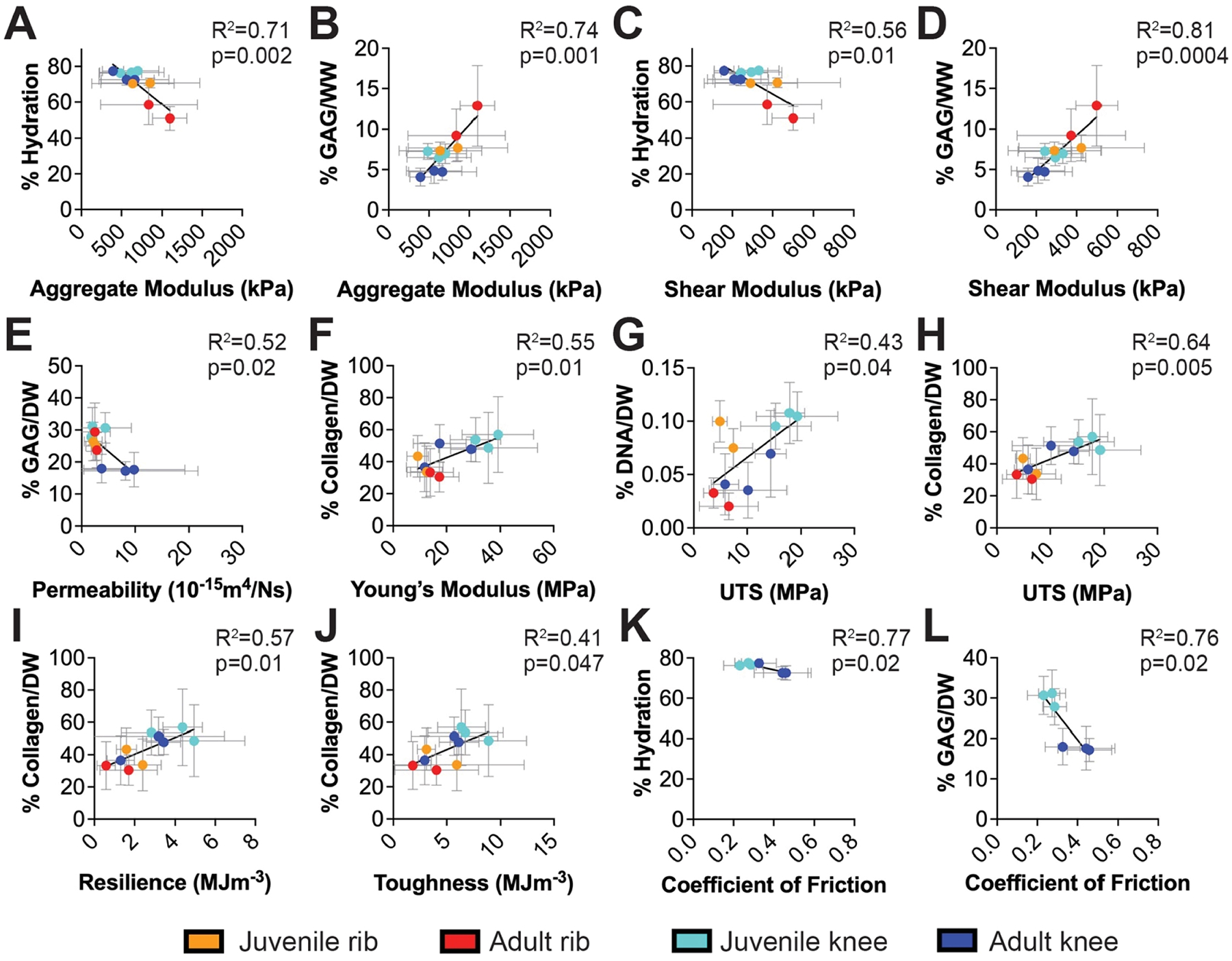
Correlations between biochemical and mechanical data sets. Pearson correlations were run between biochemical and mechanical data sets. The statistically significant correlations are included in the above figure, with, in general, compressive properties (A-E) correlating to GAG content and hydration, tensile properties (F-J) correlating to collagen content, and tribological properties (K-L) correlating to GAG and hydration.

**Fig. 8.**
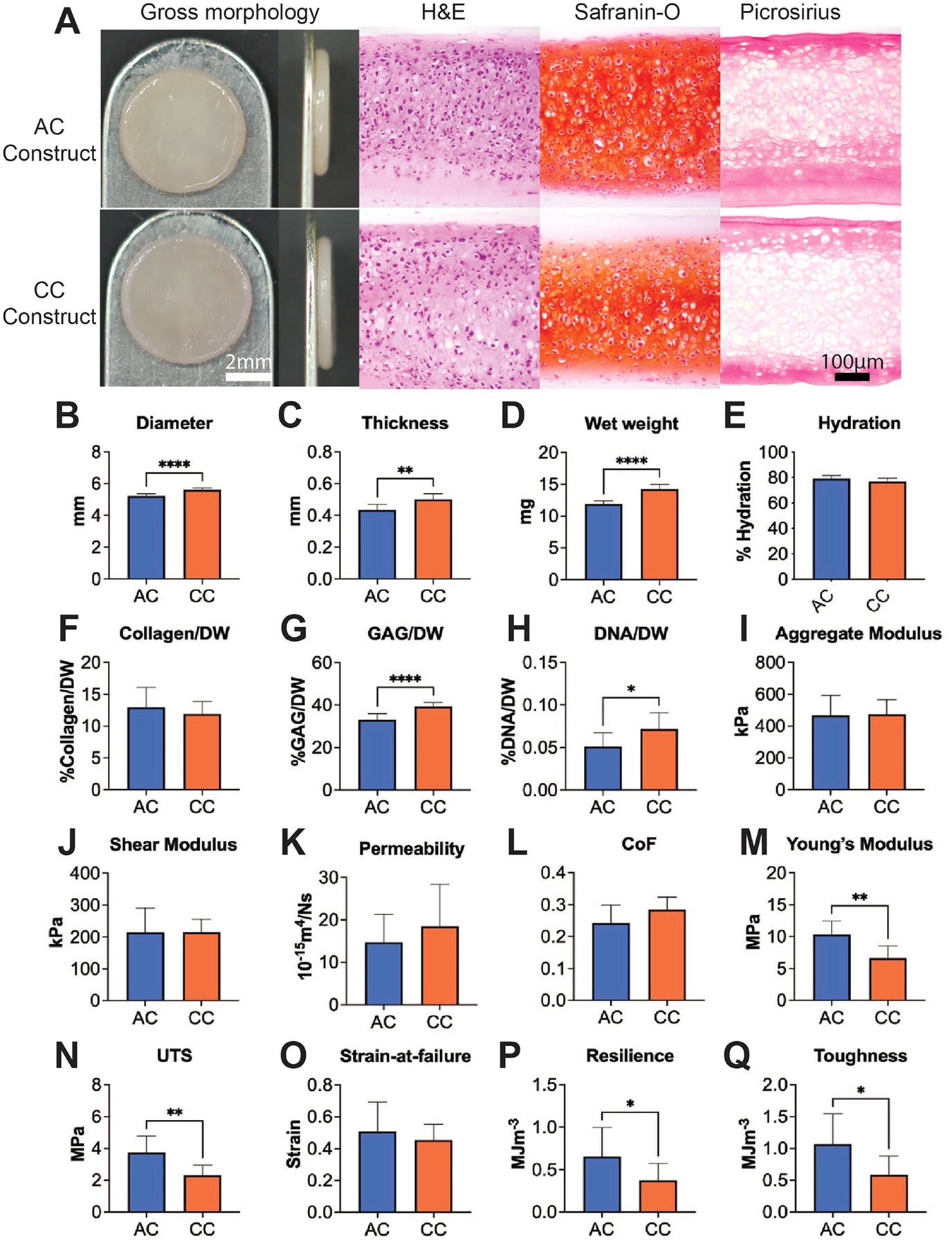
Construct morphology, histology, biochemistry, and mechanics. Gross morphology of AC and CC constructs revealed that both constructs had similar morphologic characteristics and dimensions (A-D). Tissue was stained using Hematoxylin and Eosin, Safranin-O, Picrosirius Red (scale bar = 100 μm). Biochemical contents of each group were quantified by hydration, DNA/DW, GAG/DW, Collagen/DW (E-H). Mechanically, compressive (I-K), tribological (L), and tensile (M-Q) properties were assessed. Values presented are mean+standard deviation for *n* = 6–8 with groups compared by a *t*-test (*p < 0.05, **p < 0.01, ****p < 0.0001).

The goal of this study was to identify pronounced functional differences related to anatomical structure, topographical location, or age, rather than subtle variations in mean values. Based on previous characterization studies and *in vivo* research in the minipig, [12,15] power analyses were conducted in SAS, with a significance level α of 0.05 and a power (1 – β) of 0.80 to determine the appropriate sample size for each group. For example, using the aggregate modulus as a representative outcome measure, a sample size of *n* = 6 per group, α=0.05, and standard deviation of 50 kPa, we have 80 % power to detect significant differences between the groups of at least 90 kPa. Therefore, in this study, a sample size of at least 6 was used in all groups tested. All quantitative biochemical, biomechanical, and morphological data are presented as mean+standard deviation with significance defined as *p* < 0.05. Normal distribution of the data was checked using a Jarque-Bera test, ensuring normality of the data and/or non-severe skewness or kurtosis. Native tissue characterization values (Figs. 2–5) were compared through two-way analysis of variance (ANOVA) with age and topographical location as factors followed by *t*-tests with a Holm-Sidak correction for multiple comparisons to compare juvenile vs. adult tissue at each topographical location (*n* = 6–8 per group). In the figures, the p-values listed below each graph represent statistical comparisons for various factors (e.g., age) that were calculated using two-way ANOVA. The asterisks indicating significant differences between age groups (Figs. 2–5 and Supplementary Fig. 1A) correspond to results from *post hoc t*-tests with Holm-Sidak correction for multiple comparisons. An analysis was conducted to compare average knee vs. rib values (Fig. 6) by pooling data points from all topographical locations for each age and anatomical structure (*n* = 12–48 per group); this data set was analyzed by a two-way ANOVA with anatomical structure and age as factors followed by *t*-tests with a Holm-Sidak correction for multiple comparisons to compare knee vs. rib at each age. The correlation analyses were performed using a two-tailed parametric Pearson correlation (Fig. 7). AC and CC neocartilage values (Fig. 8) were compared through a Student’s *t*-test (*n* = 7–10 per group). All statistical analyses were performed using GraphPad Prism version 10.1.1 (GraphPad Software, San Diego, California USA).

## 3. Results

### 3.1. Properties of the patellofemoral articular cartilage

Chondrocytes of the medial condyle, lateral condyle, and trochlear groove were organized with columnar stacking in the deep zone, spheroidal morphology in the middle zone, and flat morphology in the superficial zone, with 1–2 chondrocytes per lacuna (Fig. 2). The matrix stained with greater intensity in the juvenile samples, and cells appeared to be smaller in size than in the adult tissue. The degree of hydration of the samples was dependent on both age (*p* = 0.0005) and topographical location (*p* = 0.001), with juvenile tissue being more hydrated than adult. DNA/DW was significantly affected by age (*p* < 0.0001), with on average juvenile DNA content being 211 % of the adult DNA content (Fig. 2). GAG/DW was also significantly affected by age (*p* < 0.0001), with juvenile GAG content being 170 % of the adult GAG content. Collagen/DW was not significantly affected by age or topographical location. Numeric values of all biochemical data in Fig. 2 and normalizations by wet weight are listed in Supplementary Table 1.

The coefficient of friction was significantly affected by age (*p* = 0.03) and topographical location (*p* = 0.02) in the anterior-posterior axis, and age (*p* < 0.0001) in the medial-lateral axis. The effect of age was particularly apparent in the medial-lateral axis, with the adult coefficient of friction being on average 186 % of the juvenile coefficient of friction. Tribological anisotropy was assessed in juvenile and adult knee tissue (Supp. Fig. 3), and the effect of orientation was significant in adult tissue (*p* = 0.0002) but not juvenile tissue. Tensile tests in the anterior-posterior axis revealed that age significantly affected Young’s modulus (*p* = 0.0001) and UTS (*p* = 0.0003), with the Young’s modulus and UTS of the juvenile cartilage being 213 % and 175 % of the adult cartilage, respectively (Fig. 3). Topographical location significantly affected UTS (*p* = 0.007), strain-at-failure (*p* = 0.002), resilience (*p* = 0.03), and toughness (*p* = 0.003) in the anterior-posterior axis. In the medial-lateral axis, age significantly affected Young’s modulus (*p* = 0.01), UTS (*p* = 0.001), resilience (*p* = 0.05), and toughness (*p* = 0.02). Topographical location significantly affected Young’s modulus (*p* = 0.04) and resilience (*p* = 0.03). In general, the tensile properties of the medial condyle were significantly lower in the adult than in the juvenile tissue. For example, Young’s modulus of the juvenile tissue was 281 % of the adult tissue in the anterior-posterior axis and 251 % of the adult tissue in the medial-lateral axis. Tensile anisotropy was assessed in juvenile and adult knee tissue (Supp. Fig. 4), and the effect of orientation was not significant for any tensile parameter. For creep indentation data, topographical location did not significantly affect aggregate modulus, shear modulus, or permeability, and age significantly affected only shear modulus (*p* = 0.02) (Fig. 3). The adult medial condyle had a lower aggregate modulus (*p* = 0.01) and shear modulus (*p* = 0.001) than the juvenile condyle. For stress-relaxation data, age had a significant effect on 10 % relaxation (*p* = 0.001), 10 % instantaneous (*p* < 0.0001), 20 % relaxation (*p* = 0.0007), and 20 % instantaneous (*p* < 0.0001) moduli (Supp. Fig. 1). Numeric values of all biomechanical data in Fig. 3 and stress-relaxation data are listed in Supplementary Table 1.

### 3.2. Properties of the costal cartilage

CCs were organized in clusters with 3–4 cells per lacuna throughout the tissue (Fig. 4). Hydration was significantly affected by age (*p* < 0.0001), with the percent hydration of the juvenile tissue being on average 129 % of adult tissue. DNA/DW was significantly affected by age (*p* < 0.0001) and topographical location (*p* = 0.009), with the percent DNA per DW of the juvenile tissue being on average 331 % of adult tissue. GAG/DW and collagen/DW were not significantly affected by age or topographical location. Numeric values of all biochemical data in Fig. 4 and normalizations by wet weight are listed in Supplementary Table 2.

In the longitudinal axis of the rib, age significantly affected UTS (*p* < 0.0001), strain-at-failure (*p* < 0.0001), resilience (*p* < 0.0001), and toughness (*p* < 0.0001), with values of the juvenile tissue being significantly higher for each parameter. For example, the UTS of the juvenile tissue was 271 % of the adult tissue. In contrast, age did not significantly affect any tensile property in the circumferential axis of this tissue, but topographical location significantly affected UTS (*p* = 0.04) and resilience (*p* = 0.01). Tensile anisotropy was assessed in juvenile and adult rib tissue (Supp. Fig. 4). In juvenile tissue, orientation had a significant effect on Young’s modulus (*p* = 0.02). In adult tissue, orientation had a significant effect on UTS (*p* = 0.003), resilience (*p* = 0.005), and toughness (*p* = 0.003). Aggregate modulus, shear modulus, and permeability were not significantly affected by age or topographical location. For stress-relaxation data, age had a significant effect on 10 % relaxation (*p* = 0.02), 10 % instantaneous (*p* = 0.01), 20 % relaxation (*p* = 0.001), and 20 % instantaneous (*p* < 0.0001) moduli (Supp. Fig. 1). Numeric values of all biomechanical data in Fig. 5 and stress-relaxation data are listed in Supplementary Table 2.

### 3.3. Comparison of patellofemoral articular cartilage and costal cartilage

All data points were aggregated into four groups based on tissue type (i.e., patellofemoral articular cartilage or costal cartilage) and age group (i.e., juvenile or adult) to achieve a summary of the average functional properties for each tissue at each age (Fig. 6). Hydration was significantly affected by age (*p* < 0.0001) and tissue type (*p* < 0.0001), with juvenile knee tissue percent hydration being 109 % of juvenile rib tissue and the value for adult knee tissue being 135 % of adult rib tissue. DNA was significantly affected by age (*p* < 0.0001) and tissue type (*p* = 0.006), with juvenile knee tissue being 117 % of juvenile rib tissue and the value for adult knee tissue being 184 % of adult rib tissue. GAG/DW showed significant effects of age (*p* < 0.0001). Collagen/DW was not significantly affected by either factor. Young’s modulus was significantly affected by age (*p* < 0.0001) and tissue type (*p* = 0.02), with juvenile knee cartilage being 334 % of juvenile rib cartilage and adult knee cartilage being 130 % of adult rib cartilage. UTS was significantly affected by age (*p* = 0.0001) and tissue type (*p* < 0.0001), with juvenile knee cartilage being 286 % of juvenile rib cartilage and adult knee cartilage being 218 % of adult rib cartilage. Strain-at-failure was not significantly affected by either factor. Resilience was significantly affected by tissue type (*p* = 0.004), with juvenile knee cartilage being 197 % of juvenile rib cartilage and adult knee cartilage being 255 % of adult rib cartilage. Toughness was significantly affected by age (*p* = 0.006) and tissue type (*p* = 0.002), with juvenile knee cartilage being 156 % of juvenile rib cartilage and adult knee cartilage being 192 % of adult rib cartilage. Aggregate modulus was significantly affected by tissue type (*p* = 0.007), with juvenile rib cartilage being 125 % of juvenile knee cartilage and adult rib cartilage being 177 % of adult knee cartilage. Shear modulus was significantly affected by tissue type (*p* = 0.001), with juvenile rib cartilage being 124 % of juvenile knee cartilage and adult rib cartilage being 211 % of adult knee cartilage. Permeability was not significantly affected by either factor. Similarly, stress relaxation data (Supp. Fig. 1B) were significantly affected by tissue type (*p* = 0.0009 10 % relaxation modulus, *p* < 0.0001 10 % instantaneous modulus, *p* < 0.0001 20 % relaxation modulus, *p* < 0.0001 20 % instantaneous modulus).

### 3.4. Structure-function relationships

Structure-function relationships between all biomechanical and biochemical data of all data sets are shown in Fig. 7. Coefficient of friction was significantly correlated to hydration (R^2^=0.77, *p* = 0.02) and GAG/DW (R^2^=0.76, *p* = 0.02). In terms of tensile properties, Young’s modulus was significantly correlated to collagen/DW (R^2^=0.55, *p* = 0.01), UTS was significantly correlated to DNA/DW (R^2^=0.43, *p* = 0.04) and collagen/DW (R^2^=0.64, *p* = 0.005), resilience was significantly correlated to collagen/DW (R^2^=0.57, *p* = 0.01), and toughness was significantly correlated to collagen/DW (R^2^=0.41, *p* = 0.047). In terms of creep indentation data, aggregate modulus was significantly correlated to percent hydration (R^2^=0.71, *p* = 0.002) and percent GAG per WW (R^2^=0.74, *p* = 0.001), shear modulus was significantly correlated to hydration (R^2^=0.56, *p* = 0.01) and GAG/WW (R^2^=0.81, *p* = 0.0004), and permeability was significantly correlated to GAG/DW (R^2^=0.52, *p* = 0.02). Stress-relaxation correlations were found with hydration and GAG content (Supp. Fig. 2). Specifically, the 10 % and 20 % instantaneous moduli were significantly correlated to percent hydration (R^2^=0.68, *p* = 0.004 and R^2^=0.65, *p* = 0.005, respectively) and percent GAG/WW (R^2^=0.93, *p* < 0.0001 and R^2^=0.92, *p* < 0.0001, respectively). Likewise, the 10 % and 20 % relaxation moduli were also significantly correlated to percent hydration (R^2^=0.75, *p* = 0.001 and R^2^=0.85, *p* = 0.0001, respectively) and percent GAG per WW (R^2^=0.78, *p* = 0.0007 and R^2^=0.82, *p* = 0.0003, respectively).

### 3.5. Comparison of self-assembled neocartilage generated from ACs and CCs

Evaluation of the gross morphology of AC and CC constructs revealed that both had a round, flat shape (Fig. 8). No notable differences were observed in histologic staining between AC and CC constructs. The diameter of the CC constructs was significantly greater than that of the AC constructs (*p* < 0.0001), with the diameter of the CC constructs being 108 % of the diameter of the AC constructs. The thickness of the CC constructs was 115 % of the AC constructs thickness (*p* = 0.001), and the wet weight of the CC constructs was 119 % of the AC constructs wet weight (*p* < 0.0001). No differences were observed in terms of hydration or collagen/DW. The GAG/DW and DNA/DW of the CC constructs were 119 % (*p* < 0.0001) and 140 % (*p* = 0.02) of the AC constructs GAG/DW and DNA/DW, respectively. No significant differences were coefficient of friction values. AC constructs had significantly higher tensile values in terms of Young’s modulus (*p* = 0.001, 157 % of CCs), UTS (*p* = 0.002, 162 % of CCs), resilience (*p* = 0.045, 175 % of CCs), and toughness (*p* = 0.02, 181 % of CCs). No significant differences were observed in aggregate modulus, shear modulus, or permeability. Numeric values of all biochemical and biomechanical data in Fig. 8, biochemical normalizations by wet weight, and stress relaxation data are listed in Supplementary Table 3.

Functionality index values for AC and CC constructs were calculated two ways: 1) using the average adult knee functional properties from the medial condyle, lateral condyle, and trochlear groove (i.e., values displayed in Fig. 6) as native tissue comparators and 2) using the adult medial condyle functional properties (i.e., values displayed in Fig. 3) as native tissue comparators. When comparing to the knee as a whole (i.e., an average of all topographical locations assayed in the adult knee), the functionality index of AC constructs was 0.54, and the functionality index of CC constructs was 0.44. When specifically comparing to the adult medial condyle, the functionality index of AC constructs was 0.63, and the functionality index of CC constructs was 0.49.

## 4. Discussion

Given that the FDA has recognized the minipig as a common and accepted preclinical model and provided guidance on the use of ACPs, [19] this study performed a comprehensive characterization (including four biomechanical tests) of knee articular cartilage and costal cartilage by age and topography in the Yucatan minipig and evaluated tissue-engineered constructs derived from juvenile chondrocytes of both sources. The hypothesis that functional properties of these tissues would be dependent on anatomical structure, topographical location, and age was supported. Specifically, when comparing the native rib and knee cartilages, knee cartilage displayed higher tensile properties (e.g., the Young’s modulus of juvenile knee cartilage being 334 % of juvenile rib cartilage) while rib cartilage displayed higher compressive properties (e.g. the aggregate modulus of adult rib cartilage being 177 % of adult knee cartilage). Therefore, in general, knee cartilage is stiffer in tension but softer in compression. Age dependency was found for biochemical and biomechanical properties; for example, in the medial condyle, there was a 64 % decrease in Young’s modulus, a 44 % decrease in compressive aggregate modulus, and a 42 % decrease in GAG/DW in adult compared to juvenile cartilage. Topographic differences were also observed within the minipig knee (e.g., the Young’s modulus in the anterior-posterior axis of adult trochlear groove cartilage was 241 % that of adult medial condyle cartilage) but, with few exceptions, not for the costal cartilage. Structure-function relationships identified that compressive and tribological properties were correlated to hydration and GAG content (e.g., R^2^=0.74 for GAG/WW and aggregate modulus correlation), while tensile properties were correlated to collagen content (e.g., R^2^=0.64 for collagen/DW and UTS correlation). Additionally, the hypothesis that ACs and CCs could generate self-assembled neocartilage with appropriate functional properties was supported (achieving functionality index values of 0.54 and 0.44, respectively, when normalized to the knee as a whole). The coefficient of friction of CC-generated neocartilage was comparable to native knee articular cartilage (e.g., 0.28 CCs construct vs. 0.36 adult lateral condyle in anterior-posterior axis). Overall, these data will enable the development of minipig ACPs intended to repair the knee by providing gold-standard native tissue characterization values, investigating structure-function relationships of rib and knee cartilages, and comparing neocartilage generated from both ACs and CCs.

Self-assembled neocartilage derived from juvenile ACs and CCs exhibited functionally robust mechanical properties, indicating that either cell type could be used for the generation of ACPs for repair of articular cartilage of the knee. CCs have been previously identified to be an advantageous cell source due to ease of harvest, proliferative capacity, ability to redifferentiate after passaging, and capacity to generate hyaline cartilage tissue [1,33,34]. Additionally, while CCs do not natively express lubricin, culture methods have been developed to induce lubricin expression, [35] thereby reducing the coefficient of friction of CC-derived neocartilage and improving their suitability for applications in articulating joints. Costal cartilage has been used in autologous reconstruction surgeries, and obtaining healthy donor tissue from the rib is logistically advantageous to the knee and, therefore, using CCs as a cell source may facilitate the translation of cartilage repair products. Here, the capacity of ACs and CCs to generate self-assembled neocartilage was compared and evaluated using recently described release criteria for self-assembled neocartilage: 1) the construct must be morphologically appropriate (i.e., round, flat, and without cysts) and 2) the construct must have an acceptable functionality index, [10] which is a weighted average of the salient quantitative functional characteristics of the constructs normalized to values derived from the native tissue intended to be repaired [10]. The purpose of comparing AC- and CC-derived neocartilage was to directly evaluate their functional properties to inform cell source selection for future cell-based therapeutics to replace articular cartilage. Here, the AC and CC neocartilage constructs are comparable in terms of morphology and tissue organization. For example, the functionality index values of the AC and CC constructs were 0.63 and 0.49 when normalized to the adult medial condyle (the most clinically relevant target tissue in this study). For context, successful repair of TMJ using constructs with a functionality index of 0.42 has been previously reported [12]. Constructs from both sources have coefficient of friction values (0.24–0.28) on par with adult knee cartilage (e.g., the medial femoral condyle was 0.25 in the anterior-posterior axis). These data suggest that, despite functioning in drastically different native environments in the body, the two cell types might be interchangeable in the clinical setting, and our hypothesis that both AC- and CC-derived neocartilages would exhibit properties sufficient to satisfy release criteria for ACP development is supported. Overall, the morphology and functionality of constructs derived from both cell sources appear appropriate for repairing knee cartilage, but CCs may have an advantage from a translational perspective due to tissue sourcing.

Importantly, the adult minipig knee functional cartilage properties found in the current study recapitulate those of the adult human knee. For example, in a recent characterization of healthy human femoral condyles (age 26.2 ± 5.6 years), aggregate modulus values were found to range from 379–777 kPa, [15] which is in line with the adult minipig knee aggregate modulus values reported here, from 394–666 kPa. In addition, full-thickness Young’s modulus values were reported to range from 7.0–30.7 MPa [15] in comparison to 11.3–31.1 MPa in the adult minipig knee. Biochemically, GAG/DW ranged from 18.2–20.9 % [15] in the human compared to 17.1–17.9 % in the adult minipig. Collagen/DW ranged from 47.1–78.4 % [15] in the human compared to 36.5–51.4 % in the adult minipig. Cartilage’s coefficient of friction values have been reported to range from 0.001–0.6, [15,36–40] depending on the testing parameters used (e.g., counterface material, testing speed). Since the coefficient of friction is dependent on these parameters, a true comparison can only be made if a standard tribometer is operated in the same conditions across samples [28]. In a study that used the same tribological testing parameters as the current study, the coefficient of friction of human knee cartilage was reported to range from 0.22–0.26 [15], compared to 0.25–0.53 in the adult minipig in the current study. In the FDA’s guidance document for the repair of cartilage in the knee, a large animal model (e.g., minipig, horse, goat, sheep) has been described for preclinical studies to support an investigational new drug (IND) application [41]. The minipig has emerged as a promising model for cartilage tissue regeneration due to its many advantages as a model for cartilage repair [13]. The characterization data generated in the current study, confirming that the minipig knee cartilage has analogous functional properties to the human knee, further support the use of the minipig as a preclinical model for cartilage repair.

This study demonstrated that the functional properties of cartilage in both the knee and rib are dependent on age. Interestingly, the medial condyle had significant functional differences between juvenile and adult tissue in terms of tensile Young’s modulus and compressive aggregate modulus (e.g., the juvenile Young’s modulus was 281 % of the adult tissue in the anterior-posterior axis), but these trends were not observed in the lateral condyle and trochlear groove. This could indicate age-related degenerative changes in the medial condyle, though morphologically all tissue surfaces were deemed to be non-pathological. There was a decrease in GAG content with age across all locations examined in the knee (e.g., the juvenile GAG content being 170 % of the adult GAG content). Additionally, the coefficient of friction was higher in the adult cartilage compared to the juvenile cartilage (e.g., adult coefficient of friction was on average 186 % of the juvenile coefficient of friction in the medial-lateral orientation), indicating that there are functional differences in the knee as a function of age. There was a significant decrease in DNA content from juvenile to adult tissue in both the knee and rib tissue, suggesting a decrease in cellularity, which is consistent with previous data from the human femoral head that demonstrated that cellularity decreased as a function of age by approximately 40 % [42]. The minipig adult tissue in this study was comparable to human tissue approximately 18–25 years of age, while the juvenile tissue corresponded to approximately 10–12 years [16]. Therefore, our hypothesis that functional properties of rib and knee cartilage are dependent on age is supported, and these data provide insight into age-related functional changes in rib and knee cartilage tissues.

This study demonstrated that native minipig knee and rib cartilages exhibit functional differences. Most notably, native knee cartilage had higher tensile properties, but native rib tissue had higher compressive properties. These results are consistent with other animal models, such as the sheep, which reported that patellofemoral articular cartilage had a 146 % greater tensile modulus and a 171 % greater UTS, but costal cartilage had a 232 % greater aggregate modulus [1]. The differences in mechanical properties between these two tissues may be attributed to differences in biochemical composition. For example, the lower tensile properties in the rib could be the result of lower collagen content in the rib cartilage (i.e., the collagen content of the rib was 28.1 % lower in juvenile tissue and 29.6 % lower in adult tissue than knee cartilage of corresponding age. Additionally, hydration has been negatively correlated with compressive properties, [43] and, therefore, the lower hydration relative to knee cartilage observed in the rib tissue (76.8 % knee vs. 70.5 % rib in juvenile tissues, 74.1 % knee vs. 54.8 % rib in adult tissues) could in part contribute to the higher compressive properties observed in the rib. Therefore, in support of our hypothesis, rib and knee cartilages have distinct functional properties, highlighting the importance of characterizing cartilages and understanding structure-function relationships in different anatomical contexts.

This study identified topographical differences in the functional properties of the knee (i.e., medial condyle vs. lateral condyle vs. trochlear groove) but relatively few topographical differences in the rib. Regardless of age, the effect of topography is significant in several parameters within the knee, particularly within the tensile properties (e.g., in the anterior-posterior axis, UTS, strain-at-failure, resilience, and toughness). The trochlear groove had particularly high tensile properties compared to the condyles in the adult minipig. This corresponds with previous findings in the sheep in which it was reported that the trochlear groove UTS was 216 % that of the medial condyle [1]. The effect of topography was also significant in the values for knee hydration (*p* = 0.001) and coefficient of friction (*p* = 0.02 in the anterior-posterior axis), indicating that functional properties of the minipig knee are influenced by topography. With respect to rib cartilage, the only differences due to topography were greater cellularity in the lateral region and higher UTS and resilience in the medial region. These findings correspond to previous reports in the sheep which found greater DNA content in the tip of floating ribs (i.e., the more lateral region), and higher tensile properties in the mid region of the rib cartilage (i.e., the more medial region) [1]. Our hypothesis that topographical differences in the functional properties of these cartilages was supported in the knee but not in the rib. Overall, this study demonstrates that knee cartilage exhibits topography-specific functional properties, unlike rib cartilage, which is relatively homogeneous.

This study evaluated correlations between mechanical properties and biochemical properties to assess cartilage structure-function relationships across multiple cartilage types (i.e., knee vs. rib) and ages (i.e., juvenile vs. adult). Of the statistically significant correlations that were observed (Fig. 7), compressive properties were correlated to hydration and GAG content, tensile properties were correlated to collagen content, and lubrication properties were correlated to hydration and GAG content. These correlations follow traditional cartilage structure-function relationship principles in which tensile properties are imparted mainly by collagen [44,45] and compressive properties are imparted by GAG and water content [43,46,47]. However, recently there has been a growing appreciation in literature for how traditional structure-function relationships in articular cartilage may not always hold true. For example, in developing bovine articular cartilage, a ~2–3-fold change in collagen content was correlated to increases in compressive properties but there was no notable change in GAG content [48]. Another example is the compressive modulus of nose cartilage being highly correlated with collagen crosslinking (e.g., R^2^=0.92, *p* = 0.002) rather than GAG content [49]. In end plate cartilage, tensile properties were better correlated to the collagen/GAG ratio (R^2^=0.58) rather than collagen alone (R^2^=0.35), implicating the role of GAGs in modulating tensile properties [50]. The structure-function relationships identified in the current study were probed over a varied data set (i.e., multiple tissue types and ages), which may have enabled the detection of classical structure-function relationships while studies that focus on a more targeted tissue set do not observe the same trends. Therefore, future studies are needed to examine cartilage structure-function relationships across a spectrum of cartilage types, comparing both aggregated and tissue-specific trends.

Toward the translation of biologic cartilage repair products, topics such as alternative manufacturing methods and translational challenges should be considered. The constructs generated within the current study were treated with a biochemical stimulation regimen of TGF-β1, c-ABC, and LOXL2, as described in the methods section. However, the functional properties of the neocartilage could be further modulated with other stimuli including mechanical stimulation or other biochemical factors. For example, mechanical stimuli that have been shown to improve the biomechanical properties of neocartilage include fluid-induced shear stress, [51] tension, [52] hydrostatic pressure, [53,54] and the combination of mechanical and biochemical regimens [55,56]. These stimuli could be used to tune the functional properties to a particular indication. Additionally, alternative cell sources, such as auricular chondrocytes, [57] nasal chondrocytes, [58] and stem cells [59–61] may be considered for the fabrication of neocartilage implants. Therefore, there are numerous potential avenues of investigation to develop cartilage implants to treat a range of clinical indications. The translation of biologic cartilage repair products faces numerous obstacles, ranging from practical scientific issues such as implant fixation to broader considerations including intellectual property management, as recently reviewed [13,62,63]. A critical factor in the clinical translation of cell-based products is ensuring that implants can be manufactured at scale with consistent functional properties. For allogeneic implants such as those described in this study, the establishment of well-characterized cell banks offers a promising strategy to enhance scalability and reproducibility by enabling multiple product batches to be generated from a defined and validated cell source [10]. To progress through regulatory agencies such as the FDA, release criteria for the biologic product need to be defined (e.g., all implants must have an aggregate modulus between 250 kPa and 500 kPa). While these release criteria are defined by researchers on a case-by-case basis, it is important to consider what factors are quantifiable and indicative of product quality early on within the research process.

In conclusion, this was the first study to conduct a functional characterization of juvenile and adult minipig knee cartilage, rib cartilage, and self-assembled neocartilage derived from ACs and CCs. By doing so, this study provides gold standard design criteria for tissue-engineered implants intended to repair the knee using the preclinical minipig model. Properties of minipig cartilages were shown to be comparable to native human cartilages, confirming the minipig as a suitable preclinical model for products intended to repair or regenerate articular cartilage. For the generation of minipig ACPs, this study demonstrates that both CC and AC cell sources can be used to generate self-assembled neocartilage intended to repair hyaline articular cartilage by comprehensively comparing the biochemical, compressive, tensile, and frictional properties of the engineered neocartilage against native cartilage.

## Supplementary Material

1

Supplementary material associated with this article can be found, in the online version, at doi:10.1016/j.actbio.2025.12.023.

## Data Availability

The raw data files associated with this article are available on Mendeley Data at doi: 10.17632/fb432kfnfr.1.
