# Supplementary material for: Minipig costal and knee cartilage structure-function relationships and their use as cell sources for tissue-engineered analogous cellular products for cartilage repair
Source: Acta Biomater. Author manuscript; Available in PMC 2026 Jul 2. (PMC13327037; doi:10.1016/j.actbio.2025.12.023)
Supplement: 1 [file NIHMS2187893-supplement-1.docx]

**Supplementary material**

**
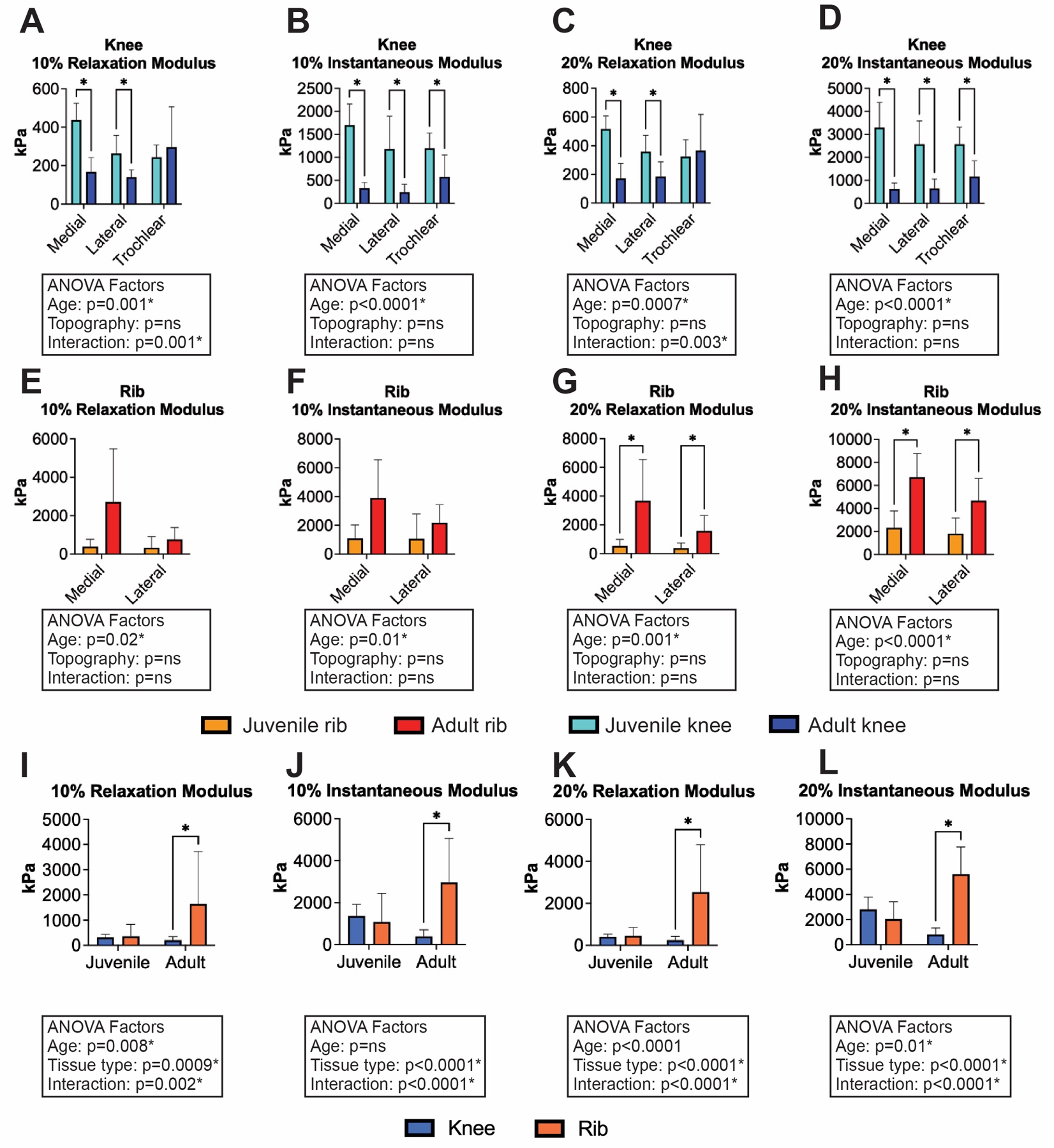
Supplementary Figure 1** Stress relaxation data. **A.** Mechanical samples were taken from the knee (medial condyle, lateral condyle, and trochlear groove) and rib (medial and lateral aspects) of juvenile and adult cartilage, and stress relaxation properties were assessed. Values presented are mean+standard deviation for n=6-8. **B.** Summary of tissue-specific (i.e., knee vs. rib) and age-specific (i.e., juvenile vs. adult) stress relaxation trends. All topographical data points were pooled to obtain an overall representation for each tissue type (i.e. juvenile rib, adult rib, juvenile knee, adult knee). Values presented are mean+standard deviation. Statistical analyses for both panels were conducted through a two-way ANOVA followed by t-tests with a Holm-Sidak correction for multiple comparisons (*p<0.05).


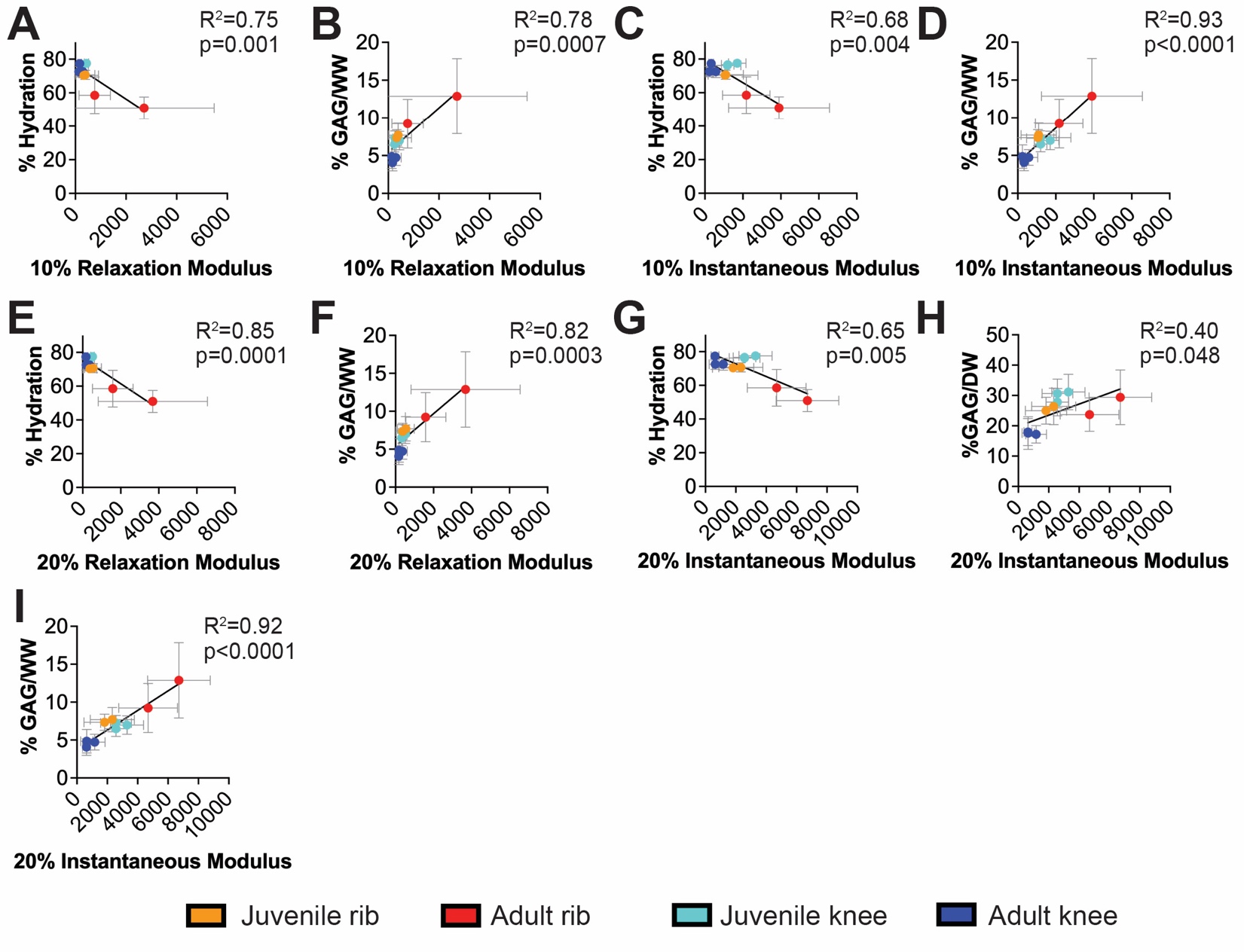


**Supplementary Figure 2** Correlations between biochemical and stress relaxation data sets. Pearson correlations were run between biochemical and stress relaxation data sets. The statistically significant correlations are included in the above figure.

**
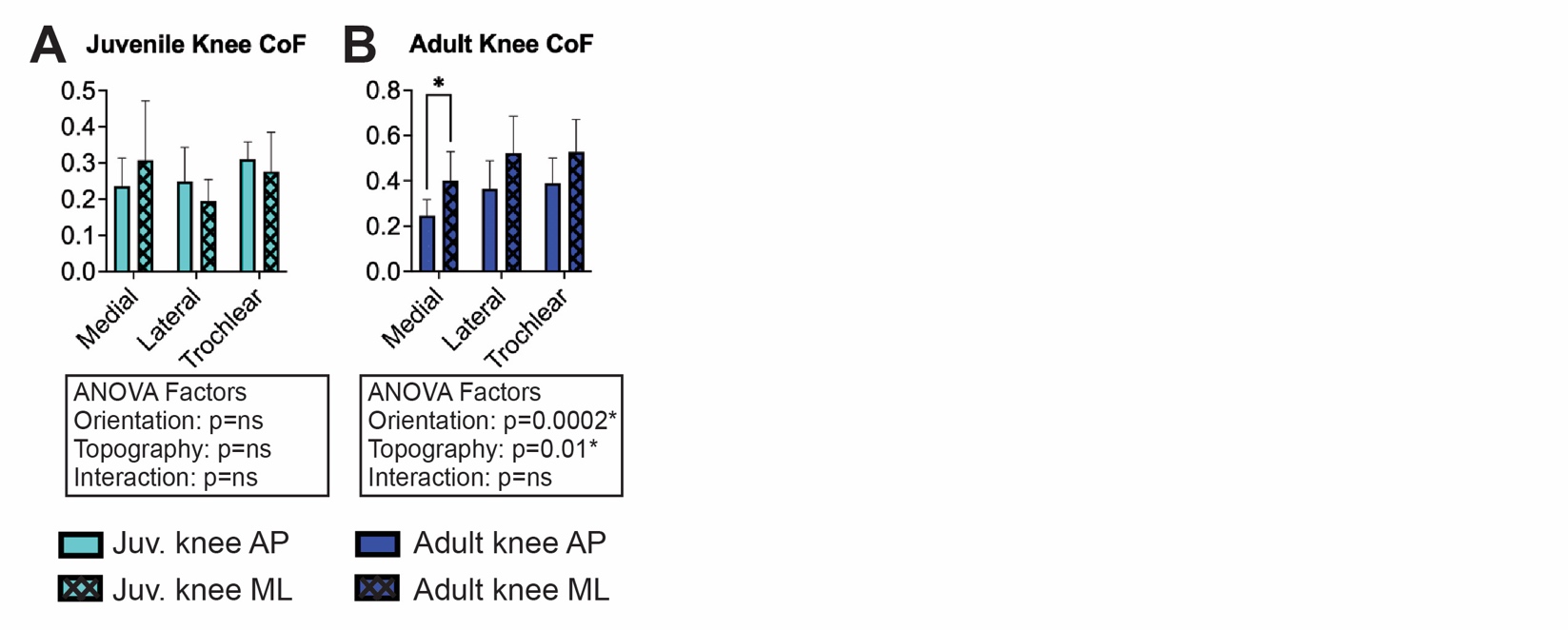
**

**Supplementary Figure 3** Tribological anisotropy of juvenile and adult knee cartilage. Samples were taken from the medial condyle, lateral condyle, and trochlear groove of juvenile and adult knee tissue and tribological properties were assessed in two orientations (solid bars = anterior-posterior axis, crosshatch bars = medial-lateral axis). Values presented are mean+standard deviation for n=6-8. Statistical analyses were conducted through a two-way ANOVA followed by an assessment of anisotropy at each topographical location using t-tests with a Holm-Sidak correction for multiple comparisons (*p<0.05).

**
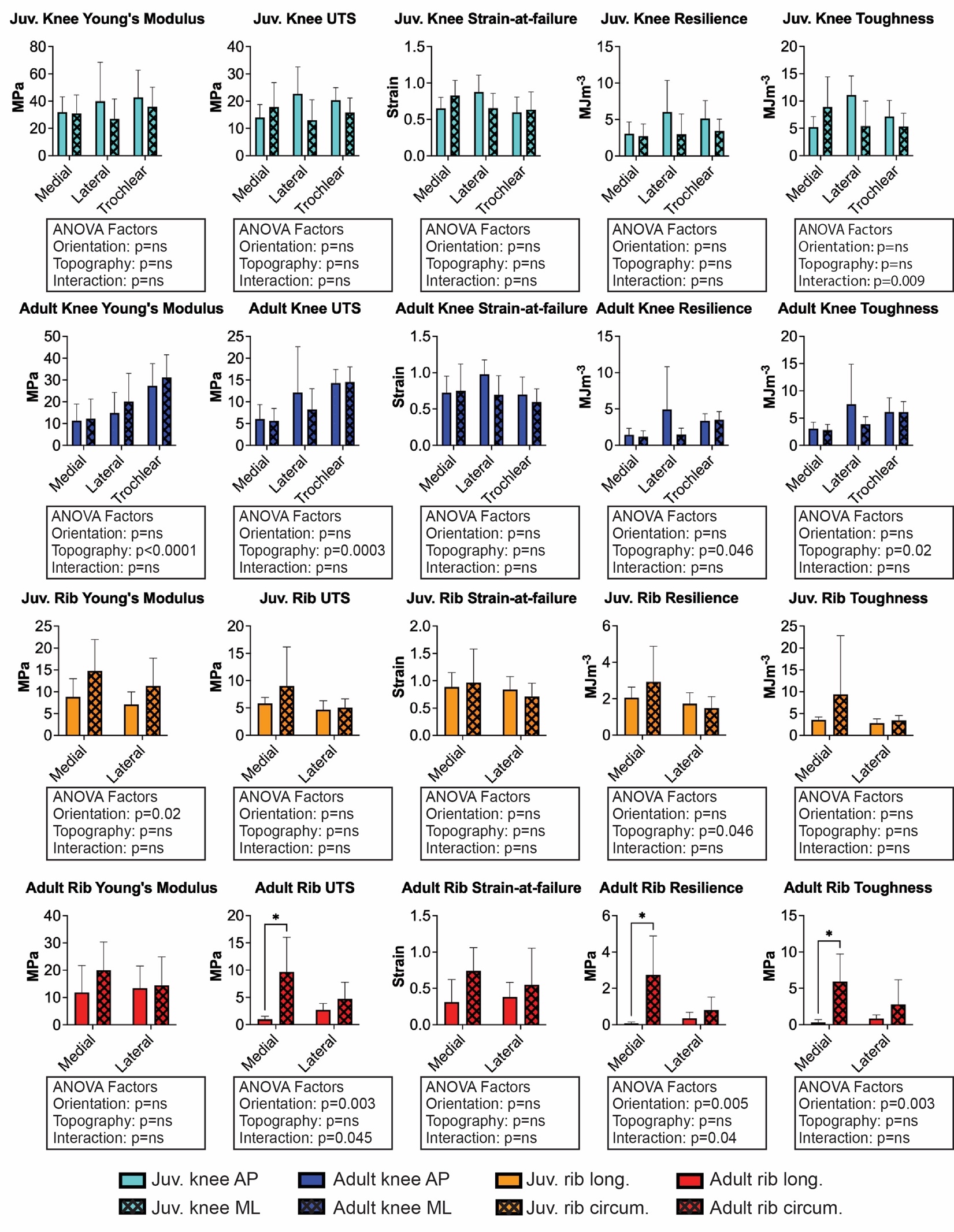
**

**Supplementary Figure 4** Tensile anisotropy of juvenile and adult knee and rib cartilage. Samples were taken from the knee (medial condyle, lateral condyle, and trochlear groove) and rib (medial and lateral aspects) of juvenile and adult cartilage, and tensile properties were assessed in two orientations (in knee: solid bars = anterior-posterior axis, crosshatch bars = medial-lateral axis, in rib: solid bars = longitudinal axis, crosshatch bars = circumferential axis). Values presented are mean+standard deviation for n=6-8. Statistical analyses were conducted through a two-way ANOVA followed by an assessment of anisotropy at each topographical location using t-tests with a Holm-Sidak correction for multiple comparisons (*p<0.05).

**Supplementary Table 1.** Comprehensive table of knee characterization values. Abbreviations: DW = dry weight, WW = wet weight, DNA = deoxyribonucleic acid, GAG = glycosaminoglycan, UTS = ultimate tensile strength, AP = anterior-posterior axis, ML = medial-lateral axis

|  | **Juvenile** |  |  | **Adult** |  |  |
| --- | --- | --- | --- | --- | --- | --- |
|  | Medial | Lateral | Trochlear | Medial | Lateral | Trochlear |
| **Biochemistry** |  |  |  |  |  |  |
| Hydration (%) | 77.5 ± 1.2 | 76.2 ± 1.3 | 76.6 ± 1.6 | 77.3 ± 1.8 | 72.6 ± 3.1 | 72.6 ± 3.5 |
| DNA/DW (%) | 0.1 ± 0.02 | 0.1 ± 0.02 | 0.11 ± 0.03 | 0.04 ± 0.03 | 0.04 ± 0.03 | 0.07 ± 0.04 |
| GAG/DW (%) | 31.1 ± 5.8 | 30.7 ± 4.7 | 27.8 ± 4.5 | 17.9 ± 4.5 | 17.6 ± 5.4 | 17.2 ± 2.9 |
| Collagen/DW (%) | 53.7 ± 13.8 | 48.6 ± 22.2 | 57 ± 23.7 | 36.6 ± 15.2 | 51.4 ± 11.8 | 47.8 ± 7.7 |
| DNA/WW (%) | 0.021 ± 0.005 | 0.025 ± 0.004 | 0.025 ± 0.005 | 0.009 ± 0.007 | 0.01 ± 0.007 | 0.018 ± 0.01 |
| GAG/WW (%) | 7 ± 1.2 | 7.3 ± 1 | 6.5 ± 1 | 4.1 ± 1.1 | 4.8 ± 1.6 | 4.7 ± 1 |
| Collagen/WW (%) | 12.1 ± 3.4 | 11.7 ± 5.7 | 13.2 ± 5.1 | 8.2 ± 3.3 | 14 ± 3 | 13.1 ± 3 |
|  |  |  |  |  |  |  |
| **Compression** |  |  |  |  |  |  |
| Aggregate modulus (kPa) | 702 ± 264 | 486 ± 267 | 626 ± 398 | 394 ± 132 | 564 ± 344 | 666 ± 419 |
| Shear modulus (kPa) | 331 ± 100 | 243 ± 133 | 295 ± 151 | 159 ± 49 | 209 ± 133 | 242 ± 138 |
| Permeability (10^-15m^4/Ns) | 2 ± 0.5 | 4.4 ± 4.9 | 1.8 ± 0.5 | 3.7 ± 4.4 | 9.8 ± 9.4 | 8.2 ± 13.5 |
| 10% Relaxation modulus (kPa) | 438 ± 87 | 264 ± 93 | 244 ± 64 | 168 ± 75 | 140 ± 39 | 296 ± 210 |
| 10% Instantaneous modulus (kPa) | 1703 ± 460 | 1180 ± 714 | 1202 ± 331 | 330 ± 124 | 246 ± 171 | 577 ± 474 |
| 20% Relaxation modulus (kPa) | 517 ± 91 | 359 ± 113 | 324 ± 117 | 173 ± 104 | 186 ± 102 | 366 ± 253 |
| 20% Instantaneous modulus (kPa) | 3297 ± 1093 | 2572 ± 1012 | 2573 ± 740 | 630 ± 256 | 649 ± 404 | 1170 ± 680 |
|  |  |  |  |  |  |  |
| **Tension** |  |  |  |  |  |  |
| Young's modulus, AP axis (MPa) | 31.9 ± 11.3 | 39.9 ± 28.7 | 42.7 ± 20.1 | 11.3 ± 7.6 | 14.9 ± 9.5 | 27.3 ± 10.2 |
| UTS, AP axis (MPa) | 13.9 ± 4.8 | 22.6 ± 9.9 | 20.3 ± 4.6 | 6.1 ± 3.3 | 12.1 ± 10.5 | 14.3 ± 3.2 |
| Strain-at-failure, AP axis | 0.65 ± 0.15 | 0.88 ± 0.23 | 0.6 ± 0.21 | 0.72 ± 0.23 | 0.98 ± 0.2 | 0.7 ± 0.24 |
| Resilience, AP axis (MJm^-3) | 3 ± 1.6 | 6 ± 4.3 | 5.1 ± 2.5 | 1.4 ± 0.9 | 4.9 ± 5.9 | 3.4 ± 1 |
| Toughness, AP axis (MJm^-3) | 5.2 ± 1.9 | 11.1 ± 3.5 | 7.2 ± 2.9 | 3.1 ± 1.2 | 7.5 ± 7.4 | 6.1 ± 2.6 |
| Young's modulus, ML axis (MPa) | 30.9 ± 13.6 | 26.9 ± 14.8 | 35.9 ± 14.2 | 12.3 ± 8.9 | 20.1 ± 13 | 31.1 ± 10.4 |
| UTS, ML axis (MPa) | 17.9 ± 9 | 13 ± 7.5 | 15.8 ± 5.4 | 5.6 ± 2.8 | 8.2 ± 4.8 | 14.5 ± 3.5 |
| Strain-at-failure, ML axis | 0.83 ± 0.21 | 0.66 ± 0.2 | 0.63 ± 0.24 | 0.75 ± 0.37 | 0.69 ± 0.27 | 0.6 ± 0.18 |
| Resilience, ML axis (MJm^-3) | 2.7 ± 1.7 | 3 ± 2.8 | 3.4 ± 1.6 | 1.2 ± 0.8 | 1.5 ± 0.9 | 3.5 ± 1.1 |
| Toughness, ML axis (MJm^-3) | 8.9 ± 5.5 | 5.4 ± 4.6 | 5.4 ± 2.4 | 2.8 ± 1.1 | 3.9 ± 1.4 | 6.1 ± 1.9 |
|  |  |  |  |  |  |  |
| **Tribology** |  |  |  |  |  |  |
| CoF, AP axis | 0.24 ± 0.08 | 0.25 ± 0.09 | 0.31 ± 0.05 | 0.25 ± 0.07 | 0.37 ± 0.12 | 0.39 ± 0.11 |
| CoF, ML axis | 0.31 ± 0.16 | 0.19 ± 0.06 | 0.28 ± 0.11 | 0.4 ± 0.13 | 0.52 ± 0.16 | 0.53 ± 0.14 |

**Supplementary Table 2.** Comprehensive table of rib characterization values. Abbreviations: DW = dry weight, WW = wet weight, DNA = deoxyribonucleic acid, GAG = glycosaminoglycan, UTS = ultimate tensile strength, L = longitudinal, C = circumferential

|  | **Juvenile** |  | **Adult** |  |
| --- | --- | --- | --- | --- |
|  | Medial | Lateral | Medial | Lateral |
| **Biochemistry** |  |  |  |  |
| Hydration (%) | 70.7 ± 2.7 | 70.4 ± 2 | 51 ± 6.5 | 58.6 ± 10.9 |
| DNA/DW (%) | 0.07 ± 0.02 | 0.1 ± 0.02 | 0.02 ± 0.01 | 0.03 ± 0.01 |
| GAG/DW (%) | 26.4 ± 6 | 25 ± 4.4 | 29.4 ± 9 | 23.7 ± 5.5 |
| Collagen/DW (%) | 33.7 ± 16.1 | 43.3 ± 13 | 30.5 ± 9.5 | 33.3 ± 14.9 |
| DNA/WW (%) | 0.022 ± 0.006 | 0.03 ± 0.007 | 0.01 ± 0.006 | 0.012 ± 0.005 |
| GAG/WW (%) | 7.7 ± 1.6 | 7.3 ± 1.1 | 12.9 ± 5 | 9.2 ± 3.2 |
| Collagen/WW (%) | 10 ± 5.1 | 12.5 ± 3.6 | 15 ± 5.3 | 13.2 ± 6.1 |
|  |  |  |  |  |
| **Compression** |  |  |  |  |
| Aggregate modulus (kPa) | 857 ± 614 | 639 ± 510 | 1098 ± 211 | 840 ± 601 |
| Shear modulus (kPa) | 424 ± 310 | 291 ± 230 | 500 ± 103 | 373 ± 268 |
| Permeability (10^-15m^4/Ns) | 2.2 ± 1.7 | 2.7 ± 1.9 | 2.5 ± 2.9 | 2.8 ± 1 |
| 10% Relaxation modulus (kPa) | 386 ± 381 | 331 ± 581 | 2711 ± 2771 | 763 ± 619 |
| 10% Instantaneous modulus (kPa) | 1097 ± 930 | 1065 ± 1729 | 3906 ± 2657 | 2181 ± 1252 |
| 20% Relaxation modulus (kPa) | 541 ± 441 | 377 ± 368 | 3687 ± 2871 | 1581 ± 1067 |
| 20% Instantaneous modulus (kPa) | 2329 ± 1449 | 1822 ± 1357 | 6715 ± 2063 | 4687 ± 1932 |
|  |  |  |  |  |
| **Tension** |  |  |  |  |
| Young's modulus, L axis (MPa) | 8.8 ± 4.2 | 7.1 ± 2.9 | 11.8 ± 9.9 | 13.4 ± 8.1 |
| UTS, L axis (MPa) | 5.8 ± 1.1 | 4.7 ± 1.6 | 1 ± 0.6 | 2.7 ± 1.2 |
| Strain-at-failure, L axis | 0.89 ± 0.27 | 0.84 ± 0.24 | 0.31 ± 0.31 | 0.38 ± 0.2 |
| Resilience, L axis (MJm^-3) | 2.1 ± 0.6 | 1.7 ± 0.6 | 0.1 ± 0.1 | 0.4 ± 0.3 |
| Toughness, L axis (MJm^-3) | 3.5 ± 0.7 | 2.8 ± 1 | 0.3 ± 0.4 | 0.8 ± 0.5 |
| Young's modulus, C axis (MPa) | 14.8 ± 7.2 | 11.3 ± 6.4 | 20 ± 10.4 | 14.4 ± 10.5 |
| UTS, C axis (MPa) | 9 ± 7.1 | 5.1 ± 1.6 | 9.6 ± 6.4 | 4.7 ± 3.1 |
| Strain-at-failure, C axis | 0.97 ± 0.62 | 0.71 ± 0.24 | 0.74 ± 0.32 | 0.55 ± 0.5 |
| Resilience, C axis (MJm^-3) | 2.9 ± 2 | 1.5 ± 0.6 | 2.7 ± 2.1 | 0.8 ± 0.7 |
| Toughness, C axis (MJm^-3) | 9.4 ± 13.4 | 3.4 ± 1.1 | 5.9 ± 3.8 | 2.8 ± 3.4 |

**Supplementary Table 3.** Comprehensive table of neocartilage characterization values. Abbreviations: DW = dry weight, WW = wet weight, DNA = deoxyribonucleic acid, GAG = glycosaminoglycan, UTS = ultimate tensile strength

|  | **AC** | **CC** |
| --- | --- | --- |
| **Morphology** |  |  |
| Diameter (mm) | 5.2 ± 0.1 | 5.6 ± 0.1 |
| Thickness (mm) | 0.43 ± 0.03 | 0.5 ± 0.04 |
| Wet weight (g) | 11.9 ± 0.5 | 14.2 ± 0.8 |
|  |  |  |
| **Biochemistry** |  |  |
| Hydration (%) | 79.2 ± 2.2 | 76.9 ± 2.7 |
| DNA/DW (%) | 0.05 ± 0.02 | 0.07 ± 0.02 |
| GAG/DW (%) | 33.1 ± 2.8 | 39.3 ± 1.9 |
| Collagen/DW (%) | 12.9 ± 3.1 | 11.9 ± 2 |
| DNA/WW (%) | 0.011 ± 0.004 | 0.017 ± 0.006 |
| GAG/WW (%) | 6.9 ± 0.6 | 9.1 ± 1.1 |
| Collagen/WW (%) | 2.7 ± 0.7 | 2.8 ± 0.6 |
|  |  |  |
| **Compression** |  |  |
| Aggregate modulus (kPa) | 466 ± 127 | 475 ± 91 |
| Shear modulus (kPa) | 214 ± 77 | 215 ± 40 |
| Permeability (10^-15m^4/Ns) | 14.7 ± 6.6 | 18.5 ± 10 |
| 10% Relaxation modulus (kPa) | 43 ± 20 | 61 ± 25 |
| 10% Instantaneous modulus (kPa) | 95 ± 68 | 109 ± 49 |
| 20% Relaxation modulus (kPa) | 73 ± 29 | 115 ± 42 |
| 20% Instantaneous modulus (kPa) | 953 ± 35 | 715 ± 90 |
|  |  |  |
| **Tension** |  |  |
| Young's modulus (MPa) | 10.3 ± 2.1 | 6.6 ± 1.9 |
| UTS (MPa) | 3.7 ± 1 | 2.3 ± 0.7 |
| Strain-at-failure | 0.51 ± 0.18 | 0.45 ± 0.1 |
| Resilience (MJm^-3) | 0.7 ± 0.3 | 0.4 ± 0.2 |
| Toughness (MJm^-3) | 1.1 ± 0.5 | 0.6 ± 0.3 |
|  |  |  |
| **Tribology** |  |  |
| CoF | 0.24 ± 0.06 | 0.28 ± 0.04 |
